# The phylogenetic relationships of geoemydid turtles from the Eocene Messel Pit Quarry: a first assessment using methods for continuous and discrete characters

**DOI:** 10.7717/peerj.11805

**Published:** 2021-08-05

**Authors:** Eduardo Ascarrunz, Julien Claude, Walter G. Joyce

**Affiliations:** 1 Department of Geosciences, University of Fribourg, Fribourg, Switzerland; 2 Institut des Sciences de l’Évolution de Montpellier, UMR UM/CNRS/IRD/EPHE, Montpellier, France

**Keywords:** Messel, Phylogenetics, Morphology, Geoemydidae, Testudines

## Abstract

The geoemydid turtles of the Eocoene Messel Pit Quarry of Hesse, Germany, are part of a rich Western European fossil record of testudinoids. Originally referred to as “*Ocadia” kehreri* and “*Ocadia” messeliana*, their systematic relationships remain unclear. A previous study proposed that a majority of the Western European geoemydids, including the Messel geoemydids, are closely related to the Recent European representatives of the clade *Mauremys*. Another study hypothesised that the Western European geoemydid fauna is more phylogenetically diverse, and that the Messel geoemydids are closely related to the East Asian turtles *Orlitia* and *Malayemys*. Here we present the first quantitative analyses to date that investigate this question. We use continuous characters in the form of ratios to estimate the placement of the Messel geoemydids in a reference tree that was estimated from molecular data. We explore the placement error obtained from that data with maximum likelihood and Bayesian methods, as well as linear parsimony in combination with discrete characters. We find good overall performance with Bayesian and parsimony analyses. Parsimony performs even better when we also incorporated discrete characters. Yet, we cannot pin down the position of the Messel geoemydids with high confidence. Depending on how intraspecific variation of the ratio characters is treated, parsimony favours a placement of the Messel fossils sister to *Orlitia borneensis* or sister to *Geoemyda spengleri*, with weak bootstrap support. The latter placement is suspect because *G. spengleri* is a phylogenetically problematic species with molecular and morphological data. There is even less support for placements within the *Mauremys* clade.

## Introduction

**Figure 1:**
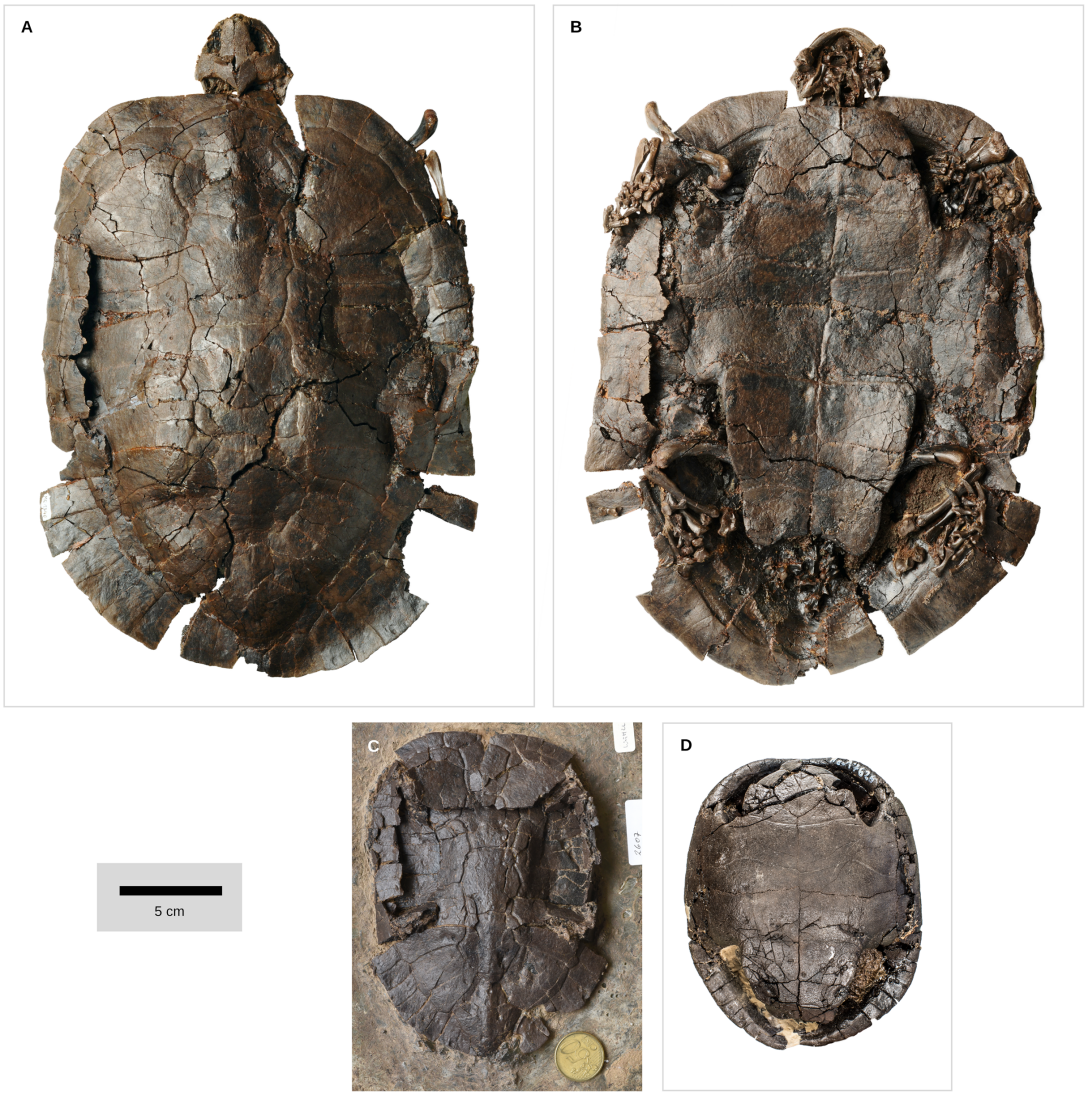
Geoemydid fossils from the Messel Pit quarry. The well-preserved *kehreri* specimen SMF ME1340 in dorsal (A) and ventral (B) views. SMF ME2607 (C) shows a common dorsal view preservation of *messeliana*, with a collapsed carapacial dome. The ventral view of HLMD ME17626 (D) displays three-dimensional preservation that is very rare for *messeliana* specimens. The carapace of that specimen is partially collapsed. Photos A and B by Anika Vogel, C by Walter G. Joyce, and D by Eduardo Ascarrunz.

The Messel Pit quarry from the middle Eocene (Lutetian, MP11) of Hesse, Germany, has yielded an abundant and well-preserved turtle fauna, including the podocnemidid *Neochelys franzeni*, and the trionychians *Allaeochelys crassesculpta* and *Palaeoamyda messeliana* (Cadena, Joyce & Smith, 2018). Staesche (1928) also described two abundant (*N* >> 100) geoemydid morphs from Messel, which are of similar shape but have clearly different sizes: “*Ocadia” messeliana* (carapace length of 10–20 cm) and “*Ocadia” kehreri* (carapace length of 25–30 cm) (Fig. 1). Several specimens of the latter have even preserved internal organs that were identified as oviducts, possibly swollen from bearing eggs (Gaßner et al., 2001). Despite this seemingly propitious material, the systematics of the Messel geoemydids is not well understood (only for convenience and simplicity, we shall refer to the Messel morphs simply as *messeliana* and *kehreri* to circumvent the nomenclatural disagreements described in what follows). The global phylogenetic relationships of extant geoemydids began to become significantly clearer in the early 2000s thanks to phylogenetic analyses of molecular data (Honda et al., 2002; Honda, Yasukawa & Ota, 2002; Spinks et al., 2004; Sasaki et al., 2006). Further work stabilised the valid species and internal relationships of major clades (Barth et al., 2004; Feldman & Parham, 2004; Le, McCord & Iverson, 2007; Spinks et al., 2012), and decisively subsumed the extant species of the genus “*Ocadia*” into *Mauremys* (*e.g*. Barth et al., 2004; Feldman & Parham, 2004; Spinks et al., 2004). Several palaeontological analyses have since conducted phylogenetic analyses that exploit the signal from molecular data (Joyce & Lyson, 2010; Naksri et al., 2013; Vlachos et al., 2019; Garbin, Böhme & Joyce, 2019; Vlachos, 2020), but similar progress on Western European geoemydid material, including the Messel material, remains wanting.

**Figure 2:**
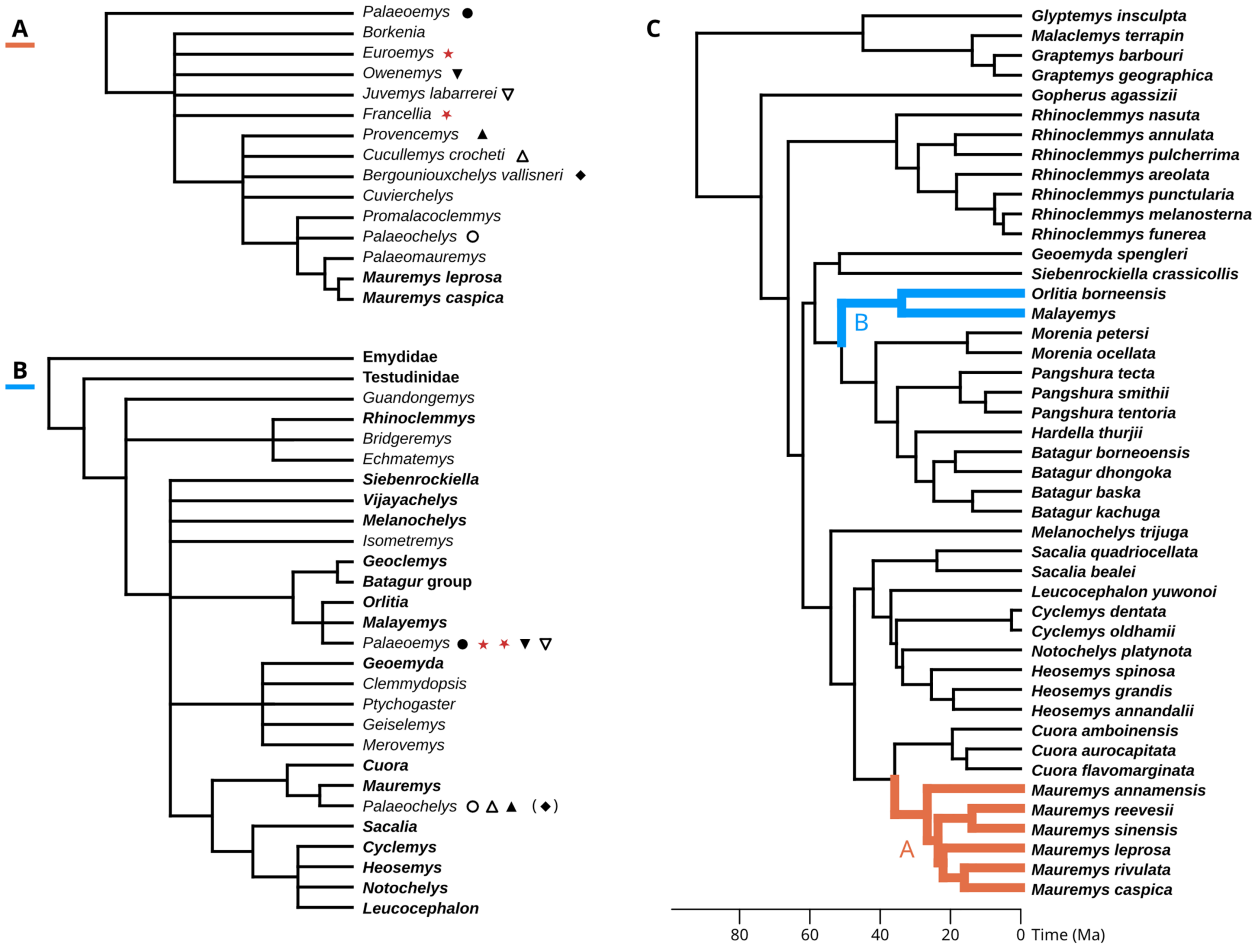
Phylogenetic hypotheses for the fossil geoemyidid turtles. (A) “Hypothesis A” from Hervet (2003, 2004), and (B) “Hypothesis B” from Claude et al. (2012). (C) shows possible placements for the Messel geoemydids in the molecular phylogeny of Pereira et al. (2017) (we only show the species as included in our analyses), following hypothesis A (in red) or hypothesis B (in blue). The symbols indicate which genera or species recognised by Hervet (2004) were synonymised by Claude & Tong (2004); the parentheses around the rhombus for *Bergoniouxchelys* indicate that it was no longer considered a synonym of *Palaeochelys* in Claude et al. (2012). The geoemydid morphs analysed in this study, *kehreri* (star) and *messeliana* (inverted star) appear in red. Extant species and clades appear in boldface.

Hervet (2003, 2004) carried out the most comprehensive systematic treatment of Western European geoemydids to date. Therein, she erected new genera for the Messel geoemydids, creating the combinations “*Euroemys kehreri”* and “*Francellia messeliana”*. In each genus she also described new species (Hervet, 2004), namely “*Euroemys vidalenci”* from the middle Eocene (Lutetian, MP12-13) of Trotte-Cos in Aude, France, and “*Francellia salouagmirae”* from the early Eocene (Ypresian, MP7) of Rians in Var, France. Hervet’s study was done under the assumption of a close relationship between *Mauremys caspica* and *Mauremys leprosa* (the only two extant geoemydid species that Hervet recognised in Europe) and a large number of European fossil geoemydids that include *kehreri*, *messeliana*, and species that historically had been attributed by various authors to *Palaeochelys*, “*Ocadia”*, and *Palaeoemys*, among others, but excluding “ptychogasterids”. That putative clade (Fig. 2A) was referred to as the “*Palaeochelys sensu* lato–*Mauremys*” group, a concept based on previous ideas of de Broin (1977; de Lapparent de Broin, 2001). Unfortunately, the phylogenetic analyses of Hervet (2003) only included the “*Palaeochelys sensu* lato–*Mauremys*” group and the outgroups *Elkemys australis*, a testudinoid from the early Palaeocene (Shanghuan) of Guandong, China, with possible but uncertain affinities to early testudinids or geoemydids (Danilov, Claude & Sukhanov, 2012), and *Platysternon megacephalum*, an extant testudinoid more closely related to emydids (Parham, Feldman & Boore, 2006; Pereira et al., 2017). Therefore, the relationships between the extensive European fossil geoemydid fauna and extant geoemydids (other than *Mauremys caspica* and *Mauremys leprosa*) were completely unexamined in the phylogenetic analysis, and the support for the “*Palaeochelys sensu* lato–*Mauremys*” group as a clade remained unclear.

In their description of testudinoids from the Eocene of Saint-Papoul, France, Claude & Tong (2004) also provided a systematic review of western European fossil geoemydids with a drastically different perspective. In general, they considered invalid various species and genera erected by Hervet (2004), and proposed that *messeliana* and *kehreri* might be conspecifics at different growth stages, and assigned them to genus *Palaeoemys* under the combination “*Palaeoemys messeliana”*. Their concept of *Palaeoemys* also includes *Palaeoemys hessiaca* (the generic type) from the middle Eocene (Lutetian) of Borken, Germany, and *Palaeoemys testudiniformis* and *Palaeoemys corroyi* from the early Eocene (Ypresian) of England and France, respectively. Claude & Tong (2004) were sceptical of the reliability of morphological characters for inferring the global phylogenetic relationships of geoemydids, and opted for taking advantage of the molecular phylogenies that had been recently published (Spinks et al. (2004), in particular) to guide their interpretation of character polarities and construct a phylogenetic scenario, which was later extended with new findings (Claude et al., 2012). In their preferred phylogenetic scenario, *Palaeoemys* (including the Messel geoemydids) is grouped in a clade with *Malayemys*, *Geoclemys*, and *Orlitia* (Fig. 2B). Although benefiting from a wider phylogenetic scope, their hypothesis has not been evaluated by less subjective means.

Our aim in this study is to determine the phylogenetic position of *kehreri* and *messeliana* in the context of the global diversity of extant geoemydids. Our results can have a wider significance for the understanding of the phylogenetic relationships of western European geoemydids because, although the position of the Messel morphs alone would not suffice to validate the entire “*Palaeochelys sensu* lato–*Mauremys*” hypothesis (hereafter “hypothesis A”) or the hypothesis of Claude et al. (2012) (which we term “hypothesis B”), it has the potential to refute important aspects of either or both.

In a previous study, some of us we found that traditional discrete morphological characters do not suffice for the reliable inference of phylogenetic relationships among extant geoemydids (Garbin, Ascarrunz & Joyce, 2018), and in another, we obtained similar results with continuous characters in the form of 3D coordinates of homologous landmarks (Ascarrunz, Claude & Joyce, 2019). However, it was possible to use the same data to make more reliable inferences about the position of individual species on a fixed reference tree, a procedure known as “phylogenetic placement” (Matsen, Kodner & Armbrust, 2010; Berger & Stamatakis, 2010). Furthermore, as the sets of anatomical features described with the discrete and continuous characters are not fully overlapping, there is potential for improving the reliability of our inferences by the combination of the two sources of characters. We thus constructed a hybrid dataset for this study, and used it in conjunction with phylogeny estimated in a recent and comprehensive molecular phylogenetic analysis (Pereira et al., 2017) to estimate the phylogenetic placement of *kehreri* and *messeliana*.

As in previous studies (Garbin, Ascarrunz & Joyce, 2018; Ascarrunz, Claude & Joyce, 2019), we evaluated the performance of different phylogenetic placement methods with our data.

### Hypotheses

To evaluate the support for the different hypotheses about the placement of the Messel geoemydids (Hervet, 2003, 2004; Claude & Tong, 2004; Claude et al., 2012), we recast them in terms of the modern understanding of the relationships of extant geoemydids after Pereira et al. (2017) (Fig. 2C).

**Hypothesis A:** *messeliana* and *kehreri* are more closely related to *Mauremys caspica* and *Mauremys leprosa* than *Cuora amboinensis*.

Hervet (2003, 2004) consistently included *Mauremys caspica* and *Mauremys leprosa* in the “*Palaeochelys sensu* lato–*Mauremys*” group, and left the relationships of other extant *Mauremys* species as an open question. In the early 2000’s there was considerable uncertainty about the concept of *Mauremys*, as the species traditionally included in the genus were found forming a non-monophyletic group (Honda, Yasukawa & Ota, 2002). Eventually, the name *Mauremys* was retained by expanding it to include the genera *Chinemys* and *Ocadia* (Barth et al., 2004; Spinks et al., 2004). Hypothesis A, as stated here, is therefore more inclusive than what Hervet might have envisaged. We confidently exclude *Cuora*, the sister group of *Mauremys*, as Hervet never included any other extant species in the “*Palaeochelys sensu* lato–*Mauremys*”.

**Hypothesis B:** *messeliana* and *kehreri* are more closely related to *Orlitia borneensis* and *Malayemys* than *Morenia petersi*.

Claude & Tong (2004, p. 20) proposed a slightly different hypothesis in which *Palaeoemys* (including the *messeliana* and *kehreri* morphs) formed a clade with *Geoclemys* and *Malayemys* to the exclusion of all other geoemydids. Spinks et al. (2004) and subsequent studies (Diesmos et al., 2005; Praschag et al., 2006) consistently found support for *Geoclemys* as sister to the clade formed by *Morenia*, *Pangshura*, *Batagur*, and *Hardella*, and therefore Claude et al. (2012, p. 649) revised their phylogenetic scenario with the tree shown in Fig. 2B. Our formulation of hypothesis B closely follows the latter version. It should be noted, however, that we unfortunately lacked sufficient data to include *Geoclemys* in our analyses.

## Materials and Methods

There is disagreement on whether *kehreri* and *messeliana* represent one or two distinct species (Claude & Tong, 2004; Hervet, 2004). We performed the placement analyses under the two-species interpretation, because it represents the more complex scenario, for which we can give a fuller discussion. However, we do not wish to endorse either alternative in this contribution.

### Phenotypic data

Our data comes from the discrete character matrix of Garbin, Ascarrunz & Joyce (2018) and the 3D landmark coordinates of Ascarrunz, Claude & Joyce (2019), with some modifications. Most importantly, we made sure to have both discrete and continuous data of each species, for which we added 20 new specimens to the discrete data matrix, based on photographs and notes from the material previously studied in Ascarrunz, Claude & Joyce (2019) (Table 1). The data originally associated with *Malayemys subtrijuga* is presented here as simply *Malayemys*, because it is possible that the collection identifications of the osteological material that we studied stand in conflict with the recently revised species delimitations within *Malayemys* (Ihlow et al., 2016). In total, we compiled phenotypic data for 40 extant geoemydids, as well as 4 extant emydids and one extant testudinid that we use as outgroups.

**Table 1:** Specimens added to the discrete and continuous datasets for this study.

Specimen	Species	Discrete data	Continuous carapace data	Continuous plastron data
FMNH 224083	*Cyclemys dentata*	●	–	●
FMNH 224092	*Cyclemys dentata*	●	–	●
FMNH 224095	*Batagur baska*	●	–	–
FMNH 224097	*Batagur baska*	–	–	●
MCZR 166446	*Glyptemys insculpta*	●	●	●
MCZR 182819	*Malaclemys terrapin*	●	●	●
MCZR 1863	*Malaclemys terrapin*	–	●	●
MCZR 1870	*Malaclemys terrapin*	●	–	–
MCZR 46253	*Graptemys barbouri*	●	●	●
MCZR 46258	*Graptemys barbouri*	–	●	●
MCZR 46278	*Graptemys barbouri*	●	●	●
MCZR 6397	*Graptemys geographica*	–	●	●
PCHP 1176	*Graptemys geographica*	●	–	●
PCHP 11927	*Cyclemys dentata*	●	●	●
PCHP 11959	*Malaclemys terrapin*	●	–	●
PCHP 3952	*Cyclemys oldhamii*	●	●	–
PCHP 4738	*Mauremys leprosa*	●	●	●
PCHP 6139	*Cyclemys oldhamii*	●	●	●
PCHP 6502	*Batagur baska*	●	●	–
YPM HERR19103	*Gopherus agassizii*	●	●	●
HLMD ME20114^†^	*messeliana*	●	–	–
HLMD BE142	*kehreri*	●	–	–
HLMD BE148	*kehreri*	●	–	–
HLMD BE157	*kehreri*	●	–	–
HLMD ME10477	*messeliana*	●	–	–
HLMD ME13437	*messeliana*	●	–	–
HLMD ME13770	*messeliana*	●	–	–
HLMD ME1444	*messeliana*	●	–	●*
HLMD ME1452	*kehreri*	●	–	–
HLMD ME14749	*kehreri*	●	–	–
HLMD ME15011	*messeliana*	●	–	–
HLMD ME15033	*kehreri*	●	–	–
HLMD ME15565	*kehreri*	●	–	–
HLMD ME17626	*messeliana*	●	–	–
HLMD ME7229	*kehreri*	●	–	–
HLMD ME7960	*messeliana*	●	–	–
HLMD ME8037	*messeliana*	●	–	–
HLMD ME8051	*kehreri*	●	–	–
HLMD ME8877	*kehreri*	●	–	–
HLMD ME9051	*messeliana*	●	–	–
SMF ME10957	*kehreri*	●	–	–
SMF ME11285	*kehreri*	●	–	●
SMF ME11389	*kehreri*	●	–	●
SMF ME11558	*kehreri*	●	●	–
SMF ME1210	*messeliana*	–	●	●
SMF ME1221	*messeliana*	–	–	●
SMF ME1340	*kehreri*	●	●	●
SMF ME1458	*kehreri*	–	●	–
SMF ME1557	*kehreri*	–	–	●
SMF ME1564	*kehreri*	–	●	●
SMF ME1679	*kehreri*	–	–	●
SMF ME172	*kehreri*	●	●	–
SMF ME1782	*kehreri*	●	●	●
SMF ME1797	*kehreri*	–	–	●
SMF ME2607	*messeliana*	●	●	–
SMF ME2767	*kehreri*	–	●	●
SMF ME2776	*messeliana*	–	●	–
SMF ME3495	*kehreri*	–	–	●
SMF ME3774	*kehreri*	●	●	●
SMF ME3777	*messeliana*	●	●	●
SMF ME717	*kehreri*	●	●	–

**Note:**

† This voucher number was assigned after we had conducted our analyses. In our data files we refer to this specimen with the provisional voucher number HLMD 2015-3-221.

* Data taken from Staesche (1928).

Institutional abbreviations (all in the USA): Field Museum of Natural History (FMNH) in Chicago, Illinois, the Chelonian Research Institute (PCHP) in Oviedo, Florida, the Museum of Comparative Zoology (MCZ) in Cambridge, Massachusetts, and the Yale Peabody Museum (YPM) in New Haven, Connecticut.

The continuous phenotypic measurements of the Messel geoemydids comes from five *messeliana* and 15 *kehreri* specimens housed in the Senckenberg Museum in Frankfurt (SMF), Hesse, Germany. Discrete characters of the Messel material were coded from nine *messeliana* and 23 *kehreri* specimens from SMF and the Hessisches Landesmuseum Darmstadt (HLMD) in Darmstadt, Hesse, Germany. We also incorporated an additional series of measurements of the plastron of the holotype of *messeliana* (HLMD-ME1444) provided by Staesche (1928). That plastron was in good condition when studied, measured, and photographed by Staesche, but it is now encased in a plaster base and can no longer be examined.

We categorised the Messel specimens as either *messeliana* or *kehreri* primarily by size, while also checking other features noted by Staesche (1928) and Hervet (2003, 2004), such as the more elliptical carapacial contour and presence of lateral carapacial carinae in *messeliana*, and the more anterior position of the humeropectoral sulcus in *kehreri*. We had no disagreements with Hervet’s (2003, 2004) assignments of the specimens that we examined.

An important limitation in our current sampling is the lack other relevant fossil geoemydids (*e.g. Palaeoemys testudiniformis, Borkenia* spp.), which could improve inferences about the Messel geoemydids (Mongiardino Koch, Garwood & Parry, 2021). We intend to incorporate more such material in further studies.

#### Continuous phenotypic data

In a previous study we collected geometric shell data of a wide range of extant geoemydids in the form of 3D coordinates of homologous landmarks (Ascarrunz, Claude & Joyce, 2019). However, data from Messel geoemydid material cannot be collected in the same way, as the fossils are without exception crushed or deformed to some degree making it impossible to accurately capture their undeformed geometry (Fig. 1).

Fortunately, rather than using landmarks coordinates and superimposition techniques, we could use ratios of linear measurements of localised features. Ratios are often used in phylogenetic studies based on anatomical specimens, as they still capture some information about shape and limit the effect of taphonomic distortion of the specimens, especially when they correspond to small features rather than global shape. Furthermore, ratios do not suffer from artificially forcing the uniform and isotropic distribution of the variance over all points, as is done by standard superimposition techniques such as generalised Procrustes analysis.

**Figure 3:**
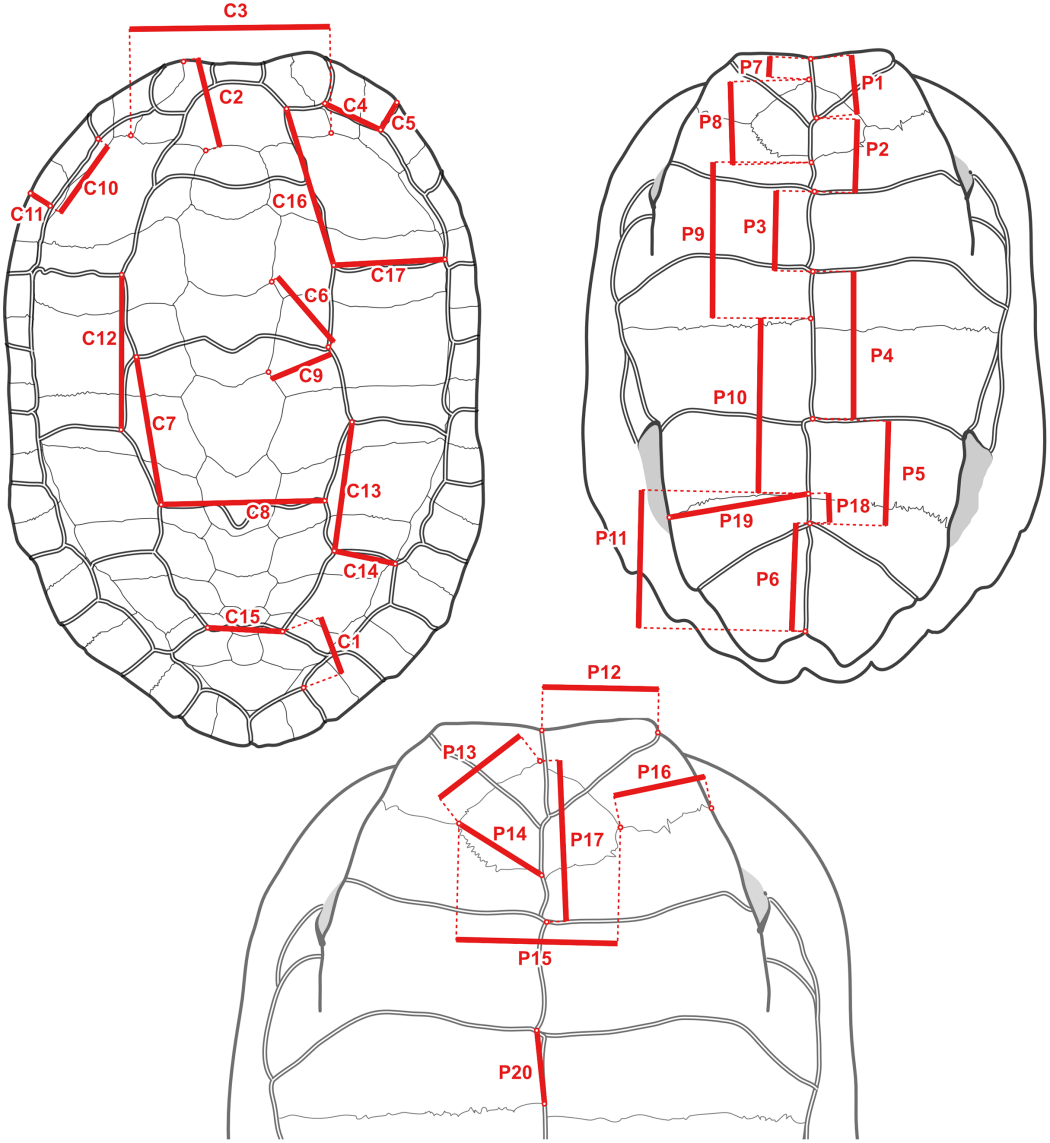
Measurements taken to construct the log-ratio characters in Table 2. On the fossil specimens measurements with a contralateral homologue were taken on whichever side they were best preserved. On specimens of extant species, the geometry of the shell was first captured with 3D landmark coordinates and symmetrised, and then the measurements were taken as distances between landmarks.

It is straightforward to compute linear measurements from the original landmark data that are strict homologues to linear distances as they would be recorded by a regular calliper with sub-millimetric precision. To do this, we defined segments that correspond to distances between chosen pairs of landmarks that we had surveyed (Fig. 3). Those segments corresponded to features that we observed to have a good chance of being reasonably well-preserved in the fossils. For instance, the length of the sulcus between the first and second pleural scutes (Fig. 3, measurement C17) or the length of the intergular sulcus (Fig. 3, measurement P1). Because such linear measurements are bound to have a strong correlation with specimen size, we implemented a rough correction by finding pairs of segments that yield ratios (Table 2) that have weak correlations (Pearson correlation coefficients between −0.3 and 0.3 in a log-log scale) with the centroid size of the carapace or the sum of the centroid sizes of the plastral lobes of the specimens of extant species. These were simple linear correlation analyses that do not take into account phylogenetic structure nor assume a model of evolution (we discuss the fit of the data to single-rate Brownian motion models often assumed in comparative methods below), and therefore do not separate phylogenetic and non-phylogenetic covariances in the data. The continuous characters used in our phylogenetic analyses are log-transformations of the ratios, or, in one case, the log-transformation of the segments (see the maximum likelihood analyses below). The log-transformation unskews the data to approximate the normal distribution assumed in Brownian motion models and normalises the effect of the arbitrary choices of numerator and denominator in the maximum parsimony analyses (Mongiardino Koch, Soto & Ramírez, 2015). We defined 10 log-ratio characters based on 17 segment characters of the carapace, and 15 log-ratio characters based on 20 segment characters of the plastron (Fig. 3). The segment characters were directly measured with a digital calliper (4 decimal digits of precision in a millimetre scale, which we rounded down to 2; similar to the precision of the microscribe that we used) on a series of SMF specimens. Segments with contralateral homologues were measured on whichever side of the specimen was best preserved. For the specimens of extant geoemydid species, the characters were measured programmatically from the 3D landmark coordinates, after steps of replicate averaging and bilateral symmetrisation with estimation of missing landmarks by reflection as described in Ascarrunz, Claude & Joyce (2019). The symmetrisation step was performed to make the measurements of extant species comparable to the measurements of the Messel material, which were not systematically taken from either side. In some cases, log-ratio characters could be obtained from multiple specimens, providing information about intraspecific variation for 227 of the 1175 cells in the matrix of continuous data. We dealt with this situation in two ways. For the main analyses with maximum parsimony (see below), we created a continuous character data matrix with the 95% confidence interval (95% CI) of the mean of the log-ratio characters assuming a normal distribution. The parsimony criterion treats ranges of continuous values analogously to polymorphic observations of a traditional ordered multi-state discrete character (Goloboff, Mattoni & Quinteros, 2006). All the analyses with other phylogenetic inference methods were performed with the point estimates of the means of the log-ratio characters, owing to limitations of their implementations. We also performed maximum parsimony analyses on the point estimates of the log-ratio means, whose results are more directly comparable to those of the other methods.

**Table 2:** Definitions of log-ratio characters used in this study.

Name	Numerator	Denominator	r	Description
RC1	C4	C5	0.000	Marginal 2, ratio of inner length to posterior width
RC2	C6	C9	0.088	Neural 3, position relative to sulcus between vertebral 2 and vertebral 3
RC3	C7	C8	0.030	Vertebral 3, ratio of lateral length to posterior width
RC4	C14	C13	0.229	Pleural 3, ratio of posterior width to inner length
RC5	C10	C11	−0.092	Marginal 3, ratio of inner length to posterior width
RC6	C15	C1	−0.185	Vertebral 5, ratio of anterior width to distance between inner vertebral 5-pleural 4 contact to inner marginal 11-marginal 12 contact
RC7	C16	C17	0.110	Pleural 1, ratio of inner length to posterior width
RC8	C12	C13	0.162	Pleural 2, ratio of inner length to anterior width
RC9	C12	C7	0.061	Ratio of inner length of pleural 2 to lateral length of vertebral 3
RC10	C2	C3	−0.180	Nuchal, ratio of length to posterior width
RP1	P1	P1+P2+P3	−0.225	Intergular sulcus, ratio to total midline length of anterior lobe scutes
RP2	P2	P1+P2+P3	0.220	Interhumeral sulcus, ratio to total midline length of anterior lobe scutes
RP3	P4	P4+P5+P6	0.176	Interabdominal sulcus, ratio to total midline length of posterior lobe scutes
RP4	P5	P4+P5+P6	0.236	Interhumeral sulcus, ratio to total midline length of posterior lobe scutes
RP5	P1+P2+P3	P4+P5+P6	0.122	Total midline length ratio between anterior and posterior lobe scutes
RP6	P17	P8	0.164	Position of humeropectoral sulcus relative to entoplastron midline length
RP7	P13	P14	0.033	Ratio between anterior to posterior lateral margins of entoplastron
RP8	P12	P1	0.046	Gular, ratio of anterior width to midline length
RP9	P11	P10+P11	−0.211	Xiphiplastron, ratio of midline length to total midline length of posterior lobe plates
RP10	P19	P11	−0.217	Xiphiplastron, ratio of anterior width to midline length
RP11	P16	P15	−0.229	Ratio of entoplastron width to length of epi-hyoplastral suture
RP12	P7	P1	0.050	Ratio between lengths of interepiplastral suture and intergular sulcus
RP13	P9	P7+P8+P9	0.063	Hyoplastron, ratio of midline length to total midline length of anterior lobe plates
RP14	P18	P11	0.212	Xiphiplastron, ratio of interfemoral sulcus to midline length of the xiphiplastron
RP15	P20	P9	0.092	Hyoplastron, ratio of interabdominal sulcus to midline length of hyoplastron

**Note:**

Refer to Fig. 3 for the measurements in the numerator and the denominator. *r* is the Pearson correlation coefficient between the log-ratio character and the log-centroid size of the carapace or the logarithm of the sum of centroid sizes of the plastral lobes.

#### Discrete phenotypic data

We built a discrete character matrix based on the matrix published in Garbin, Böhme & Joyce (2019), with the following modifications (numbers refer to characters in the original matrix). We excluded all characters that could not be coded or were not relevant for the Messel material (27, 28, 29, 55, 56, 57, 58, 60, 61, 62, 73, 75, 76, 77, 78, 79, 80, 84, 87, 90, 92, 93), and the discrete characters similar to our new ratio characters (34, 74, 81, 82, 83, 88, 89). The latter does not imply that we avoided all correlations between discrete and continuous characters, but merely that we removed the discrete characters that were glaringly redundant. Characters 48 and 49 were also excluded for partial redundancy, although they were formulated in terms of maximal dimensions that did not always meet homologous landmarks. We modified the characters pertaining to the carapacial carinae, because we observed that ontogenetic variation was confounding putative homologies (carapacial carinae tend to become less prominent or disappear with age in many species). Thus, we merged characters 1, 2, and 5, and deleted characters 3, 4, and 6. The final modified matrix has 60 discrete characters for the same 47 species as scored for the continuous data.

Character 46 made reference to the extent of the sulcus between the twelfth marginals, including a state for the condition in which the sulcus does not exist because the marginals have fused. The proposed homology relations thus implied between those states are mistaken. The extent of the intermarginal sulcus is determined by the degree to which the fifth vertebral extends posteriorly between the marginals. If the fifth vertebral were to extend back all the way to reach the posterior edge of the carapace, the intermarginal sulcus would disappear because the twelfth marginals would no longer be in contact with each other. The fusion of the twelfth marginal scutes also results in the disappearance of the intermarginal sulcus, but it is clearly a different and incompatible phenomenon that occurs prior to the appearance of the anlagen of the sulci (Cherepanov, 2006). Thus, we split character 46 into two characters: one for the fusion of the marginals, and another for the extent of the intermarginal sulcus.

Intraspecific variation was represented in almost all the characters with polymorphic coding (Campbell & Frost, 1993; Wiens, 1995), where all the states of a character observed in different conspecific specimens are included in the corresponding cell of their species. Other coding schemes also incorporate information about the frequencies of the states, but they don’t perform well with this dataset (Garbin, Ascarrunz & Joyce, 2018). Only characters pertaining the presence of carapacial carinae and marginal spikes were coded following the “any-instance” scheme (Murphy, 1993; Wiens, 1995) (*i.e*. species are coded with the most derived state observed among all specimens), because we observed that their derived states tend to be most clearly manifested in juveniles and young adults, and sometimes lost in old specimens (also reported by Claude & Tong (2004)). Our revised matrix contains 60 discrete characters, 51 of which are parsimony-informative. We refer to the discrete characters in our revised matrix as characters D1 to D60. Uninformative characters were excluded in all the analyses and parsimony scores reported here.

Characters were treated as ordered whenever there were clear intermediate states, such as elements that vary in number, size, or position along a single dimension.

### Phylogenetic methods

We used the maximum likelihood method by Revell et al. (2015) and the Bayesian method of Parins-Fukuchi (2018a) only on continuous characters, as these methods are specific for these characters. We used maximum parsimony for the analysis of the continuous and discrete characters combined. With all three methods we performed two kinds of analyses: a phylogenetic placement performance analysis with the data of extant species only, and the phylogenetic placement analysis that incorporates the fossils. We give more details about the methods below, as well as a general comparison between them in Table 3.

**Table 3:** Comparison of the phylogenetic placement methods used in this study.

Method	Mode of inference	Type of character	Clock	Character correlation correction	Weights
Locate.fossil (Phytools)	Maximum likelihood	Continuous (mean)	Strict	Orthogonal rotation	Equal
Cophymaru	Bayesian	Continuous (mean)	No	None	Calibrated against reference tree
Parsimony	Maximum parsimony	Continuous (95% CI and mean) and discrete	No	None	Equal or implied

All phylogenetic placement analyses were done with the phylogeny from Pereira et al. (2017), henceforth “the Pereira tree”. We also used the mean divergence times estimated by Pereira et al. (2017) in our maximum likelihood analyses. Maximum parsimony analyses were performed with TNT 1.5 (release of June 2020) (Goloboff & Catalano, 2016).Various other analyses and tree-related tasks were done with ape v5.3 (Paradis, Claude & Strimmer, 2004) running on R v3.6.1 and a custom phylogenetics package written by E. A. (https://github.com/eascarrunz/Phylodendron2.jl) on Julia v1.4.1. Parallelisation of time-consuming computations was managed with GNU Parallel (Tange, 2011).

### Phylogenetic placement performance analyses

In order to assess the ability of our data and methods to estimate the phylogenetic position of extinct species, we used the same data and methods to perform the same task with extant species. Assuming the Pereira tree to represent a reasonably accurate estimate of the phylogenetic relationships of extant testudinoids, we assessed the performance of phylogenetic placement of extant species with a leave-one-out procedure employed in previous studies (Garbin, Ascarrunz & Joyce, 2018; Ascarrunz, Claude & Joyce, 2019; see also Berger & Stamatakis, 2010). A species is removed from the Pereira tree and then reinserted using the only morphological data. This procedure is repeated for each extant species in the tree.

We measure the error in the placement of a species }{}i as the number }{}d\left( i \right) of nodes between the original (“correct”) position and the branch on which it was reinserted. The maximum possible value of }{}d\left( i \right) is }{}{\rm\epsilon} \left( i \right) - 1, where }{}{\rm\epsilon} \left( i \right) is the eccentricity of the node of species }{}i, *i.e*. the maximum number of nodes along the path between the node of }{}i and any other node in the tree. We compare placement errors across species as proportions of their respective maximum possible values, which is the scaled placement error }{}{d_s}\left( i \right) = {{d\left( i \right)} \over {{\rm\epsilon} \left( i \right) - 1}}. Using the scaled placement errors we characterise the accuracy of phylogenetic placements across all species in the tree as a cumulative error distribution curve, the corresponding area under the curve (AUC), and the median. When maximum parsimony identified multiple most parsimonious placements for a species, we use the median of the scaled error of those placements to construct the cumulative error distribution curve. Greater values of AUC and lower medians imply that an analysis has higher accuracy, yielding greater proportions of species placements with lower amounts of scaled placement errors. All computations of placement errors were done with unrooted trees, because the algorithms that we used work by identifying common bipartitions in the Pereira tree and the re-placement trees.

The phylogenetic placement performance analyses were done with maximum parsimony and Bayesian inference with the models and settings detailed below.

### Maximum likelihood analyses

We used the maximum likelihood phylogenetic placement approach of Revell et al. (2015) as implemented in the phytools package (Revell, 2012), which only works with continuous characters. We will refer to it as “locate.fossil”, after the name of the Phytools function in which it was implemented. In it, the characters are modelled with a Brownian process where the amount of expected morphological change in a lineage is the product of the instantaneous diffusion rate and the amount of time during which the lineage evolves (Felsenstein, 1981). The instantaneous diffusion rate is assumed to be constant over the entire tree, which amounts to a “strict clock” model. The information about the time of evolution is given in the branch lengths of the reference tree; in our case, the dated Pereira tree. Locate.fossil tries out the placement of a query species on each branch of the reference tree and optimises the branch lengths with the constraints that the divergence and tip times of the reference tree be kept constant, and that a given temporal bound for the occurrence of the query species is respected. When the query species are extant is the corresponding tip is simply set to the present (0.0 Ma), and the analysis is performed with the locate.yeti function of phytools. In the placement analyses of the Messel geoemydids the upper and lower temporal bounds passed to the locate.fossil function are 48.25 Ma and 47.41 Ma (Lenz et al., 2015).

As the reference tree topology and branch lengths are fixed and assumed approximately correct, this approach allows us to correct for the correlations between the continuous characters by rotating the data with the loadings of a phylogenetic principal component analysis (Revell, 2009). In theory, this would make the use of ratio characters unnecessary. We evaluated the performance of the method with the rotated log-segment characters, and the raw and rotated log-ratio characters.

### Bayesian analyses

We used the Bayesian Markov chain Monte Carlo (MCMC) phylogenetic placement approach introduced by Parins-Fukuchi (2018a), which was implemented by the author in the Cophymaru program (https://github.com/carolinetomo/Cophymaru, master branch commit 9f82d61). We shall refer to this approach as “Cophymaru”. It estimates the placement of one or more query species from continuous character data. Like the approach of Revell et al. (2015), it is based on a Brownian process, but they in Cophymaru the diffusion rate that governs the evolution of all the characters is allowed to vary across branches of the tree, as the branch lengths are estimated in units of expected accumulated Brownian variance rather than time. Given that the branch lengths that describe the evolutionary process under this model are not known *a priori*, it is not possible to correct for correlations between characters with the orthogonalisation procedure of Revell et al. (2015).

For the Bayesian estimation of the branch lengths we used a compound Dirichlet prior with alpha parameters equal to 1.0 and beta parameters equal to the sum of branch lengths of the tree optimised by maximum likelihood (Parins-Fukuchi, 2018a).

Cophymaru allows us to further refine the treatment of the data by computing character weights based on restricted maximum likelihood (Felsenstein, 1981), following a method introduced by Berger & Stamatakis (2010). This is done by optimising the branch lengths of the reference tree and a large collection of random trees (not including the query species to be placed on the phylogeny later). The weight of each character is derived from the fraction of times that the likelihood of the character is greater in the reference tree than in a random tree. The program makes it possible to use those fractions as the weights themselves, or to create binary weights that exclude the characters where the fraction is lower than 0.95. We evaluated the performance of Cophymaru with equal weights, binary weights, and fractional weights. We also evaluated the performance with and without the z-score data transformation used by Parins-Fukuchi (2018a). That transformation rescales the data so that all the characters have unity variance, in accordance with the shared diffusion rate assumed by the model, and ensures that there are no characters with very low variance that could cause difficulties in MCMC mixing.

In the performance analyses we ran a single MCMC of 5 million generations for each species, sampled every 1000 generations. We discarded at first 10% of the generations as burn-in, and checked chain convergence with the Geweke statistic (Geweke, 1991) using the R package coda v0.19-3 (Plummer et al., 2006). When a chain was not found to have converged, we repeated the process discarding another 10% of the generations as burn-in until the Geweke statistic was compatible with chain convergence and the effective sample size was of at least 500. Chains that failed to meet those two conditions were re-run for 10 million generations, which was sufficient for obtaining posterior samples that satisfied the same requirements. This entire process was automated. We summarised the posterior distribution of each analysis with the maximum clade credibility tree computed with SumTrees v4.4.0 (Sukumaran & Holder, 2010, 2018), which we used to compute the placement errors.

For the placement of the Messel fossils, we ran 4 MCMC of 20 million generations sampled every 10,000 generations, and assessed convergence visually with Tracer (Rambaut et al., 2018). We modified the Cophymaru program to compute the character weights with 1,000 random trees (instead of the 100 that are hard-coded in the program), and to be able to use the exact same weights in different runs. Every run was done with a separate instance of the Cophymaru program. We used the tree samples from only one run (the four runs converged in all the fossil placement analyses) and discarded the first 10% of the generations as burn-in.

### Maximum parsimony analyses

We use the term “cost scheme” to refer to the ensemble of settings that determine the costs of transitions between character states, and ultimately the total parsimony score of any given tree. Such settings include scaling, the use of unordered and ordered characters, step matrices, *a priori* or implied character weights, and so forth.

The use of continuous characters highlights the problem of scaling, *i.e*. the amount of change in a character that should be considered equivalent to a unit of change in another. This issue is particularly evident when discrete and continuous characters are combined. There is no agreed general approach to the scaling problem, although implied weights have been proposed to have limited compensatory effects on range differences (Goloboff, Mattoni & Quinteros, 2006). We explored three scaling options (Mongiardino Koch, Soto & Ramírez, 2015): (1) keeping the continuous characters in their original (log-transformed) scale, (2) scaling each continuous character to have a total range of unity, and (3) z-score standardisation (scaling each continuous character to have unit variance).

We performed phylogenetic placement analyses with equal weights and with extended implied weights (Goloboff, 1993, 2014). In the extended implied weights analyses we implemented a series of values for the concavity constant k (1, 2, 3, 6, 10, 25, 50, 75, 100, 125, 250, 500, 750, and 1,000). Artefactually lower amounts of homoplasy can be observed in characters that have more missing data, which leads to the inflation of their implied weights. Extended implied weights can compensate for that phenomenon by adjusting k for each character according to a function that extrapolates the homoplasy content of the missing entries as a proportion R of the homoplasy content in observed entries (Goloboff, 2014). We used different values of R (0.0, 0.25, 0.5, 0.75, and 1.0) in combination with each value of k in the phylogenetic placement performance analyses. Of the resulting 213 cost schemes, we selected the one that maximised AUC for use in the phylogenetic placement of the Messel geoemydids.

Placement searches involved a single random addition sequence followed by hill-climbing rearrangements by subtree pruning and regrafting and tree bisection and reconnection (the command MULT 1). That search strategy is sufficient to find optimal trees, because we are only estimating the position of one or two species. The exact branch-and-bound method is not implemented for implied weights in TNT. The support for the placement of the fossils was estimated with 5,000 bootstrap pseudoreplicates (Felsenstein, 1985).

## Results

### Placement performance analyses

#### Maximum parsimony

**Figure 4:**
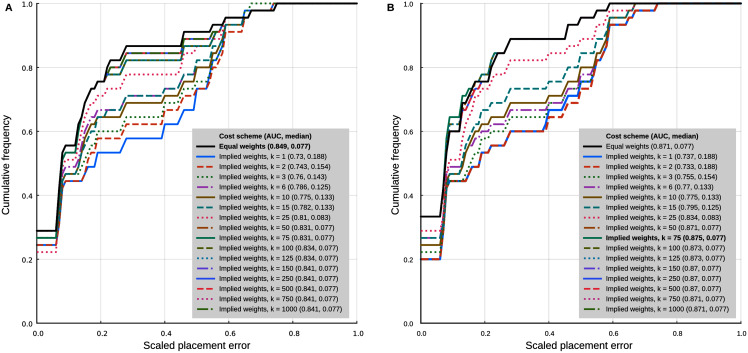
Phylogenetic placement performance with maximum parsimony with continuous and discrete characters. (A) Analysis with 95% CI ranges of log-ratio characters and equal weights. (B) Analysis with point-estimates of means of log-ratio characters and implied weights. Analysis settings discussed in the text. AUC, Area Under the Curve.

In the analyses with 95% confidence intervals (95% CI) of the log-ratio characters, the best placement performance corresponded to a cumulative placement error area under the curve (AUC) of 0.849 with a median placement error of 0.077 (Fig. 4A). Those results were obtained with the log-ratio characters scaled by z-scores and equal weights. Among all trials of combined log-ratio and discrete data, the most important factor that affected performance was the scaling of the log-ratio characters, followed by the value of *k*. Different values of the extrapolated homoplasy proportion *R* had comparatively little effect.

The separate analyses of the log-ratio and discrete characters show that the log-ratio characters carry most of the phylogenetic signal. Under the optimal cost scheme mentioned above (equal weights), the log-ratio characters alone yield a cumulative error placement AUC of 0.823 and error placement median of 0.125. The discrete characters alone under the same settings yield a maximum cumulative placement error AUC of 0.741 (median = 0.912) with equal weights. The separate analyses also show that the optimal cost schemes are not the same as when the continuous and discrete data are analysed jointly. The best performance of the log-ratio data alone (cumulative placement error AUC = 0.829, median = 0.125) was found without scaling and a *k* value of 3. The best performance of the discrete characters (cumulative placement error AUC = 0.741, median = 0.192) was found with equal weights. These results suggest that better performance in the joint analyses could be attained by setting separate *k* values for the log-ratio and discrete data partitions. We did not attempt to further optimise the cost scheme in that way because there are too many possible combinations of parameter values. We identified species that were placed with low accuracy in placement performance analyses. Only six species were placed with scaled nodal errors higher than 0.27 in the parsimony analyses with the combined dataset (z-scaled log-ratios) and optimal cost scheme (equal weights): *Mauremys annamensis* (0.58), *Geoemyda spengleri* (0.55), *Leucocephalon yuwonoi* (0.67), *Gopherus agassizii* (0.46), *Orlitia borneensis* (0.75), and *Rhinoclemmys nasuta* (0.46).

Placement performance analyses using point estimates of the means of the log-ratio characters instead of 95% CI showed similar behaviour in general, but with higher maximum performance (cumulative placement error AUC of 0.875, median = 0.077) in the joint analyses of continuous and discrete data with light implied weighting (*k* = 75) (Fig. 4B). Also, *Orlitia borneensis* had the much lower scaled nodal error of 0.25.

#### Locate.fossil

**Figure 5:**
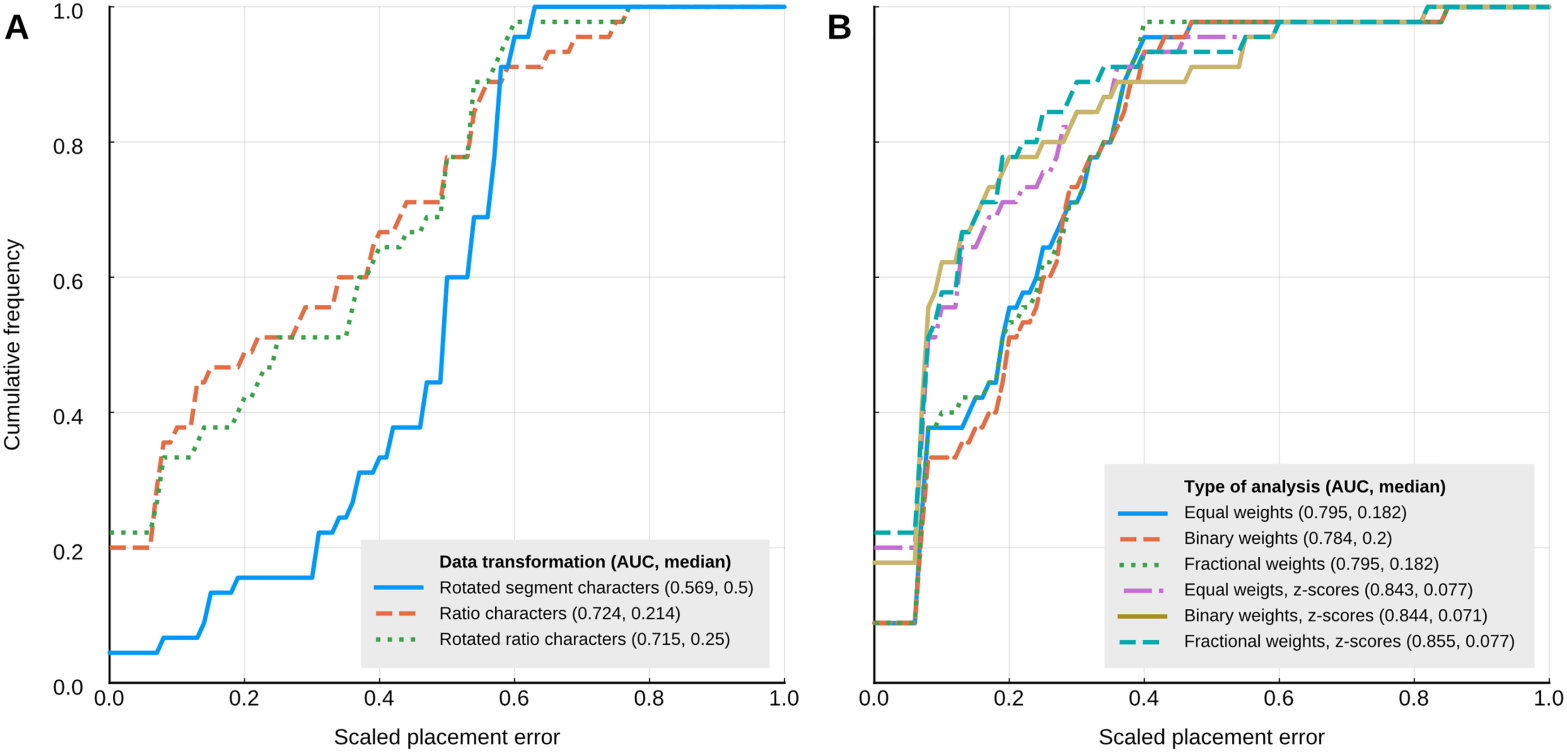
Placement performance of methods based on Brownian motion models. (A) Maximum likelihood with a strict clock model (Revell et al., 2015). (B) Cophymaru (Parins‐Fukuchi, 2018a). AUC, Area Under the Curve.

The maximum likelihood analyses with a strict clock model had a worse placement performance than the other methods that use continuous characters (Fig. 5A). The AUC of the cumulative placement error with ratio characters was 0.724 (median = 0.214), and slightly worse when the ratio characters were rotated for phylogenetic orthogonalisation (AUC = 0.715, median = 0.25). Rotated (phylogenetically orthogonalised) segment characters had very poor performance, with a cumulative error placement AUC of 0.569 and a median of 0.5.

#### Cophymaru

Cophymaru performed best with z-score transformed data (Fig. 5B). In those analyses, fractional weights showed a clear performance advantage (AUC = 0.855, median = 0.077) over equal and binary weights. In the analyses without the data transformation, equal and fractional weights performed were tied for best performance (AUC = 0.795, median = 0.182), suggesting that the optimal weighting scheme is contingent to the properties of the character data.

### Placement analyses

**Figure 6:**
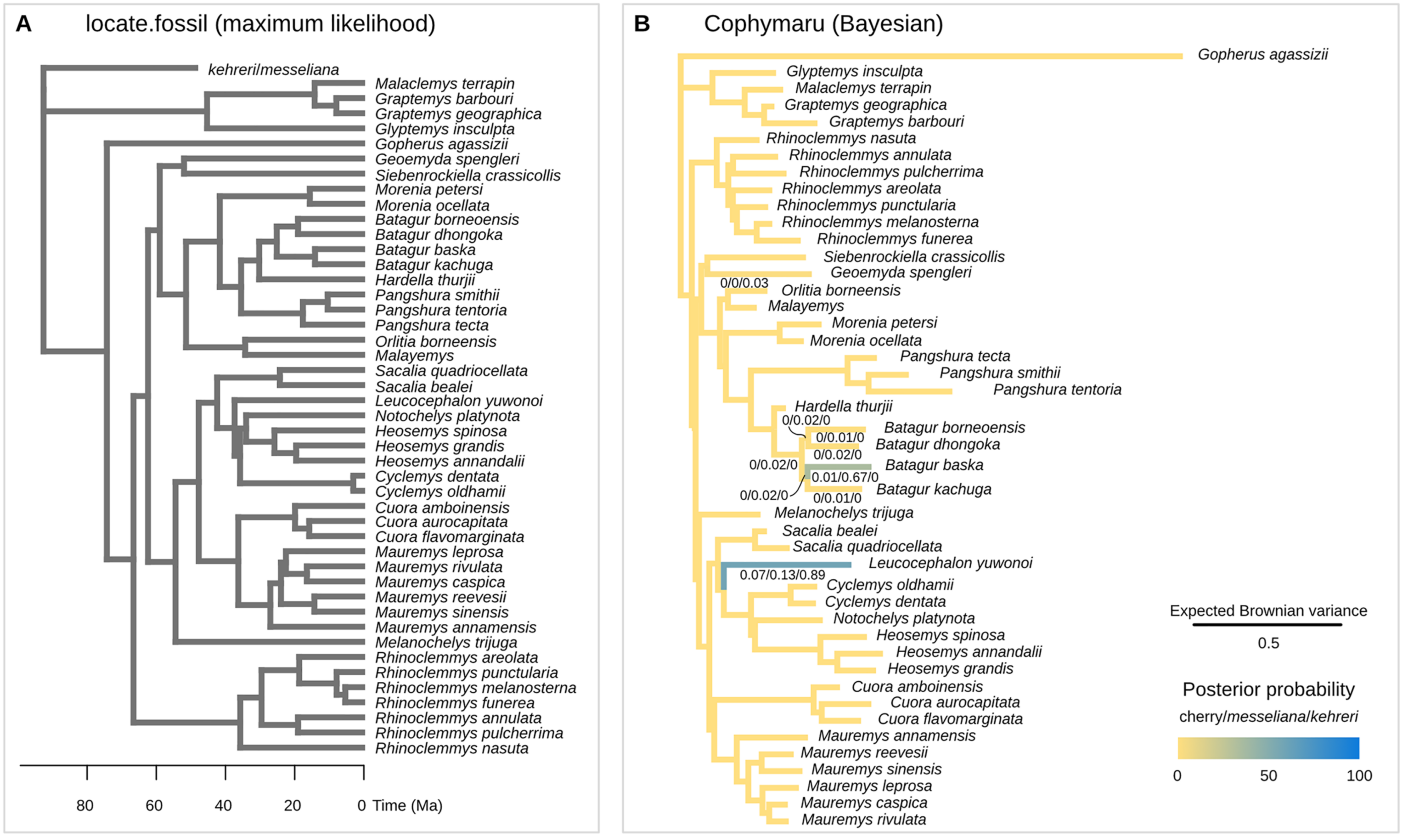
Phylogenetic placement of the Messel geoemydids estimated with methods based on models of Brownian motion. (A) locate.fossil placements with maximum likelihood with a strict clock. The maximum likelihood placement of *messeliana* is identical to the placement of *kehreri* shown here. (B) Bayesian analysis with cophymaru with unconstrained rates of evolution across branches. Colours and numbers under the branches in B indicate the posterior probability of placements on that branch. Posterior probabilities lower than 0.01 are not shown.

**Figure 7:**
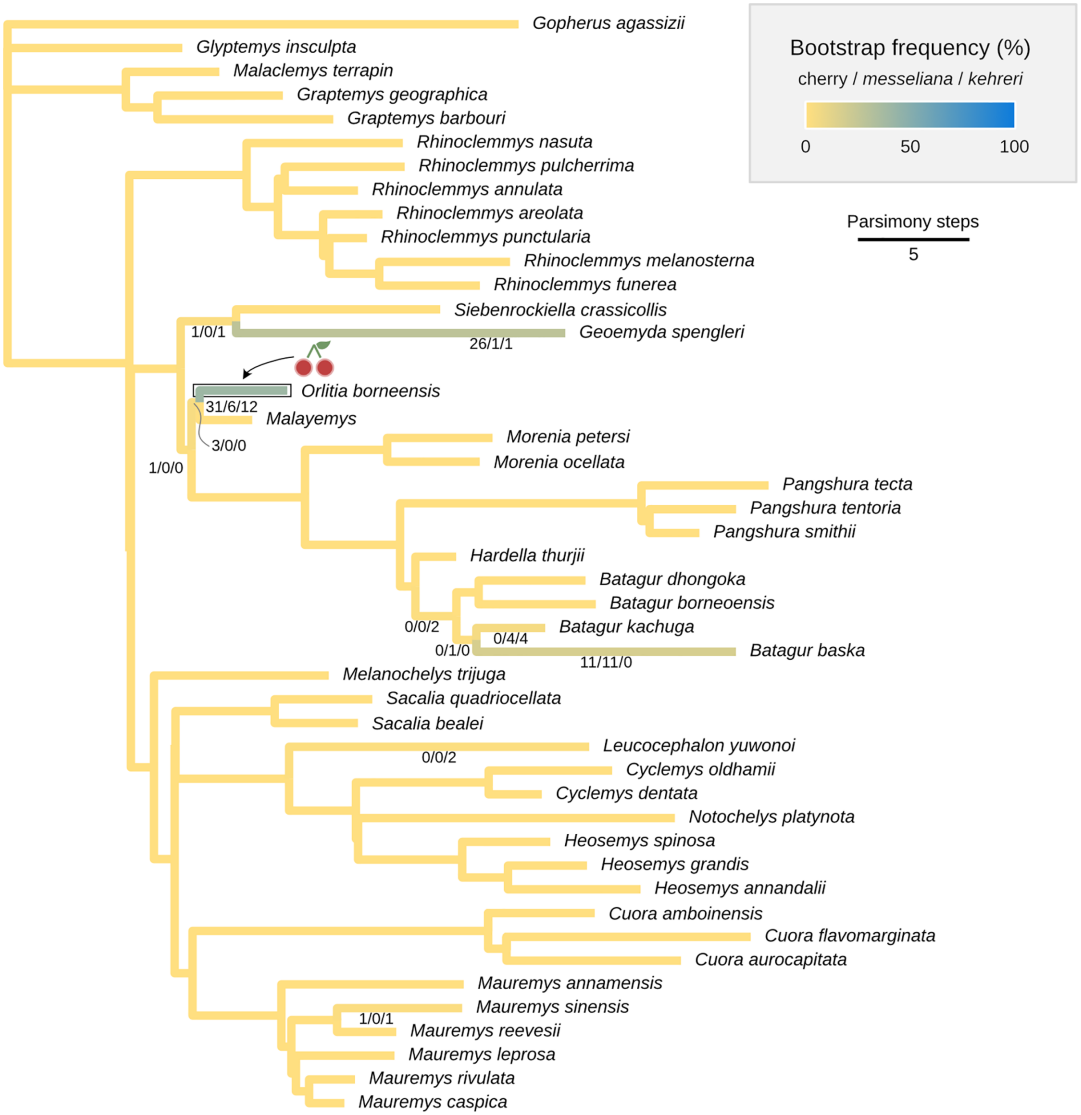
Maximum parsimony placement of the Messel geoemydids. The cherry symbol shows indicates the placement of *kehreri* and *messeliana*, sister to each other.

**Figure 8:**
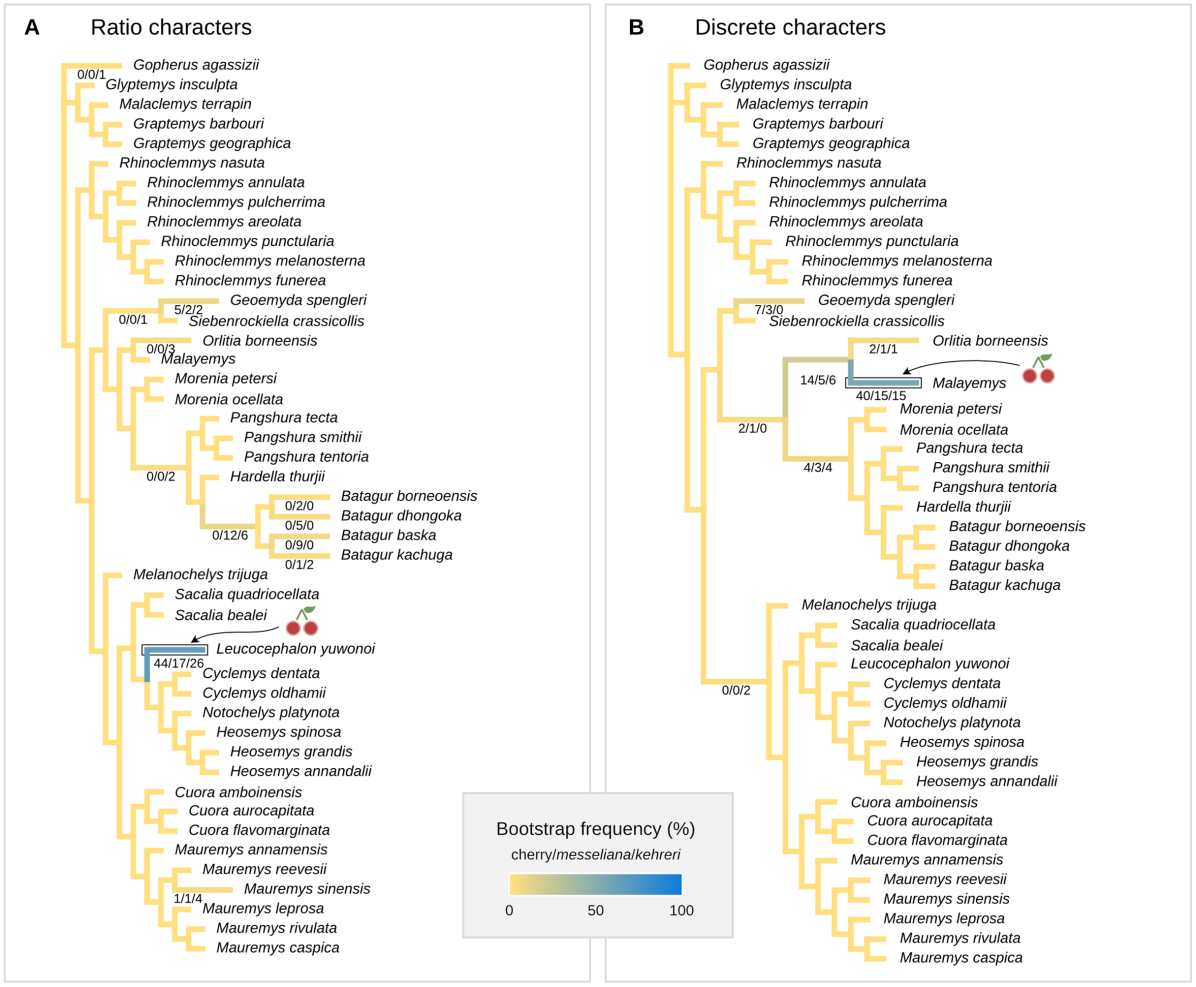
Global most parsimonious placements and placement bootstrap supports with only one type of data: either log-ratio characters (A) or discrete characters (B). For visualisation purposes, branch lengths are arbitrary and do not reflect character change.

There are three general kinds of placements of *messeliana* and *kehreri* relative to each other. In the first kind, *messeliana* and *kehreri* placed in different branches of the tree. In the second kind the two are placed in the same branch in succession, with either *messeliana* or *kehreri* closer to the root of the tree. Finally, in the third kind the two are sister to each other, forming a clade that is attached to a branch of the tree. McKenzie & Steel (2000) introduced the term “cherry” to refer to clades containing only two tips. We take advantage of that terminology to refer more specifically to the cases in which *messeliana* and *kehreri* are found being sister to each other. In the following, we present results in which the *messeliana*-*kehreri* cherry is placed on some branch of the Pereira tree. Placements of the kind 1 and 2 have negligible support in the results of most analyses, so we do not treat them in detail. In Figs. 6–8 we report the bootstrap frequency or posterior probability of the placement *messeliana* and *kehreri* separately on a branch (not forming a cherry), those values correspond to the total support for placements of kind 1 and 2 on that branch.

#### Locate.fossil

The current implementation of the method (Revell et al., 2015) cannot handle the simultaneous placement of two or more species, therefore we conducted two analyses each placing *messeliana* and *kehreri* individually. The separate analyses found the same maximum likelihood placement for *kehreri* and *messeliana*: in polytomy at the root of the tree (Fig. 6A).

#### Cophymaru

We performed the placement analyses with the z-score transformed data and fractional weights (Fig. 6B). We found strong support for the placement of *kehreri* alone as sister to *Leucocephalon yuwonoi* (posterior probability = 0.89), and found the most strongly supported placement for *messeliana* as sister to *Batagur baska* (posterior probability = 0.67), with also some support for placements sister to *Leucocephalon yuwnoi* (posterior probability = 0.13). We found low posterior probabilities for cherry placements either sister to *Leucocephalon yuwonoi* (0.07) or *Batagur baska* (0.01).

#### Maximum parsimony

We conducted the placement of the Messel geoemydids with maximum parsimony following the optimal cost schemes identified for the combined continuous and discrete data with the point estimates of the means of the log-ratio characters (z-scale, *k* = 75, any value of *R*) and the 95% confidence interval of the means of the log-ratio characters (z-scale, equal weights; notice that parsimony scores are not directly comparable between the two treatments of log-ratio characters due to different character weighting). The most parsimonious placement of the Messel geoemydids is a cherry sister to *Orlitia borneensis* with 31% of bootstrap support when 95% CIs of the log-ratio characters are used, consistent with hypothesis B with a parsimony score of 627.56 (Fig. 7). A cherry placement sister to *Geoemyda spengleri* also received moderate bootstrap support (26%). Placements sister to *Batagur baska* received low bootstrap support (10%), and placements on other branches reached no more than 5% bootstrap support.

With the means of the log-ratio characters, the global most parsimonious placements for the Messel geoemydids found *messeliana* and *kehreri* forming a cherry sister to *Geoemyda spengleri*, with a parsimony score of 7.78703 and a bootstrap support of 44%. We refer to this placement as hypothesis C. Alternative placements that received bootstrap support higher than 5% were in a cherry sister to *Malayemys* (19%), in a cherry sister to *Orlitia borneensis* (11%), and in a cherry sister to *Batagur baska* (7%).

The most parsimonious placement compatible with hypothesis A is in a cherry sister to *Mauremys annamensis* when discrete data is analysed either with 95% CIs of the means (parsimony score = 633.01, bootstrap support = 45%), or just the means of log-ratio characters (parsimony score = 7.84462, bootstrap support = 51%). The most parsimonious placement compatible with hypothesis B was a cherry sister to *Malayemys* with a parsimony score of 7.79436 and 57% of bootstrap support, based on discrete data and means of log-ratio characters.

We also performed placement analyses with only either the log-ratio or the discrete characters (Fig. 8), with their respective optimal cost schemes. With 95% CIs of log-ratio characters alone and *k* = 3, the most parsimonious placement is a cherry sister to *Leucocephalon yuwonoi* (Fig. 8A). With mean log-ratios and k = 10, and the results are similar to the Cophymaru analyses: the most parsimonious solution placed *kehreri* as sister to *Leucocephalon yuwonoi* with 29% of bootstrap support and *messeliana* as sister to *Batagur dhongoka* with 33% of bootstrap support, and low bootstrap support (11%) for a cherry placement sister to *Leucocephalon yuwonoi*. The analysis with discrete characters alone (Fig. 8B) was performed with equal weights and found the most parsimonious placement for *messeliana* and *kehreri* as a cherry sister to *Malayemys* with 40% of bootstrap support.

**Figure 9:**
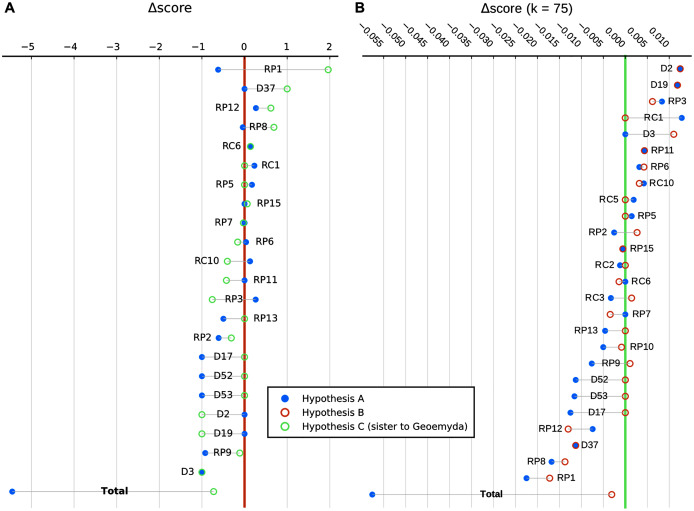
Character-wise and total parsimony score differences between the most parsimonious placement and the placements under the main hypotheses considered in this study. (A) Analysis with 95% CI ranges of log-ratio characters and equal weights. (B) Analysis with point-estimates of means of log-ratio characters and implied weights. Greater score differences reflect less homoplasy with a hypothesis relative to the most parsimonious placement. Only characters with non-zero differences in either hypothesis are shown, sorted by magnitude. RC, carapace log-ratio character; RP, plastron log-ratio character; D, discrete character.

We show in Fig. 9 a summary of each character’s contribution to the parsimony fit of the focal hypotheses relative to the most parsimonious placement and to each other. We discuss the impact of different characters in the following section.

## Discussion

### Placement methods

Our results indicate that log-ratio characters carry significant phylogenetic information for placement analyses with Cophymaru and maximum parsimony. The inclusion of discrete characters in maximum parsimony further improved placement performance. According only to the performance metrics that we evaluated, optimal placements (AUC = 0.875, median = 0.077) were obtained with maximum parsimony of discrete and mean log-ratio characters with light implied weights (*k* = 75) and z-scaling. We also assessed the performance of parsimony analyses incorporating the intraspecific variation of the log-ratio characters, which we implemented with 95% confidence intervals of the means. Although the optimal performance of these other analyses is slightly lower (AUC = 0.849, median = 0.077), we recognise that continuous intraspecific variation is important information that should not be ignored just like polymorphism in discrete characters should not be ignored (Garbin, Ascarrunz & Joyce, 2018), and that its inclusion helps to account for the effects of random sampling error. In our study, or even in general with most palaeontological data, there are limited numbers of specimens available per species, so one we should not assume high accuracy in the point estimates of the means of the log-ratio characters. Indeed, we show here that intraspecific variation of continuous characters, even when not exhaustively sampled, can have appreciable effects on the preferred phylogenetic hypotheses.

The two treatments of intraspecific variation of log-ratio characters yielded different most parsimonious placements, but both also resulted in moderate bootstrap support for cherry placements consistent with hypothesis B (close relation to *Malayemys* and *Orlitia*) and C (sister to *Geoemyda*). Considering the low error in phylogenetic placement performance and the greater inclusion of relevant data, we conclude that the maximum parsimony analyses with discrete and continuous data are the most reliable, and that hypotheses B and C are the best supported by the data. In the rest of this subsection, we also discuss the behaviour of the other placement methods, which might also be of more general interest.

Ascarrunz, Claude & Joyce (2019) found best placement performance of continuous landmark data with maximum likelihood applied to a simple Brownian motion implemented in Phylip (Felsenstein, 2005), over spatial parsimony (Catalano, Goloboff & Giannini, 2010) and squared-change parsimony (Maddison, 1991). With Bayesian phylogenetics, it is possible to design models with a Brownian process for the continuous characters and an Mkv state substitution process for the discrete characters (Lewis, 2001). We made several attempts to implement such models with the software RevBayes (Höhna et al., 2016), but our Markov Chain Monte Carlo runs displayed anomalous behaviour and therefore are not interpretable. We give a detailed description of the models and the results of those analyses in an Appendix S1.

We found that the best placement performance results of continuous data alone was with Cophymaru. The Brownian motion models of evolution implemented in Phylip and Cophymaru are almost identical (Felsenstein, 1981), but Cophymaru uses Bayesian inference and has a built-in character weighting approach.

In contrast, locate.fossil, the strict-clock maximum likelihood method of Revell et al. (2015), performed poorly (Fig. 5A). It is likely that the problem with this method is the strong assumption of a constant rate of evolution, which has been found to have a poor fit to empirical data (Chira & Thomas, 2016; Puttick, 2018). Furthermore, rate shifts are indicated by the fact that the branch lengths in units of expected Brownian variance estimated by Cophymaru clearly deviate from the branch lengths in time units of the Pereira tree (Fig. 6) and ultrametricity in general. These deviations from the fixed rate model could also have compromised the phylogenetic orthogonalisation of the characters, which is based on a phylogenetic principal components analysis with the same strict-clock model (Revell, 2009). In theory, the orthogonalisation procedure would improve placements with log-ratio characters and even make it possible to use segment characters. In practice, we did not see any improvement (Fig. 5A).

Another problematic aspect of locate.fossil is that it depends on a single reference tree with fixed branch lengths in units of time, which must come from a previous divergence time analysis where the clade or tip temporal calibrations are derived from some other data. Logically, it is impossible for a fossil to be placed in a clade that is younger than itself, regardless of the strength of the morphological signal. The compatibility of the age of the fossil with the clade ages estimated from some other data does have some evidential weight, but it is preferable to perform an analysis where the signals of the morphological and temporal data are considered jointly in the estimation of the divergence times (Heath, Huelsenbeck & Stadler, 2014; Gavryushkina et al., 2017). Support measures in those analyses would also be easier to interpret.

**Figure 10:**
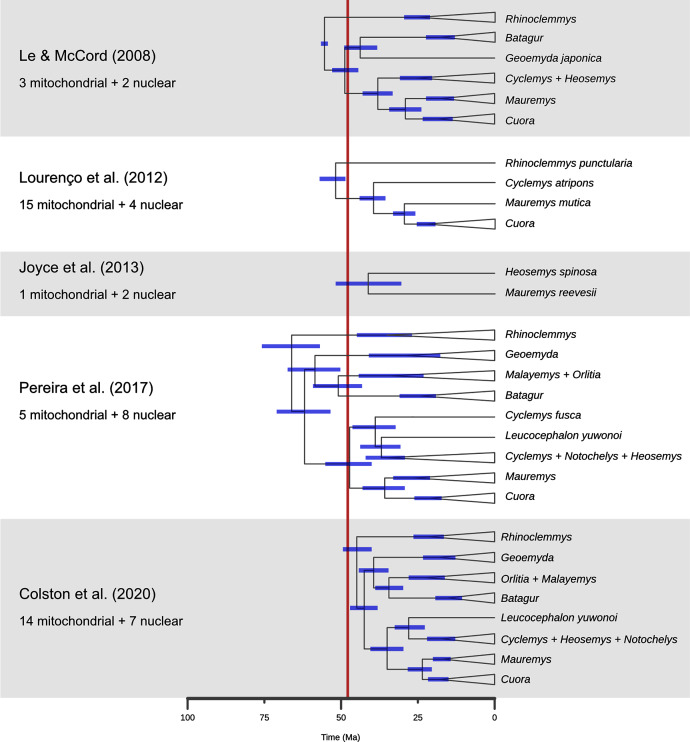
A comparison of dated phylogenies of geoemydids recovered from different studies, with the number of mitochondrial of nuclear loci that were analysed. The topologies are simplified to highlight the relationships that are more relevant to the placement analyses. The position of each node indicates the mean of the posterior distribution of ages, and the blue bars indicate its 95% highest probability density interval. The red band marks the time span for the occurrence of the Messel geoemydids. Note that *Cyclemys fusca* was found separate from the other *Cyclemys* in Pereira et al. (2017). The topology shown for Colston et al. (2020) is based on the majority rule consensus of the posterior sample of trees.

An example of the possible conflict between a dated tree and morphological signal occurs in hypothesis A. In the dated Pereira tree the mean age of the divergence between *Cuora* and *Mauremys* occurred is 43 Ma (Fig. 10), whereas the youngest limit for the age for the Middle Messel Formation is 47.41 Ma (Lenz et al., 2015). To rule out hypothesis A on those grounds alone would have been unsatisfactory.

Finally, the placement of *kehreri* and *messeliana* in a polytomy at the root of Testudinoidea likely reflects the aforementioned shortcomings of locate.fossil. Analyses of ratio characters alone with other methods favoured placements as either sister to *Leucocephalon* or *Geoemyda*. The former is chronologically impossible taking the currently available dated trees at face value (Fig. 10; Thomson, Spinks & Shaffer, 2021) and the latter yields very short branches that do not fit well the observed amounts of morphological evolution. Possible branch lengths of placements sister to *Geoemyda* could be of at most 4.08 Myr in the dated Pereira tree, and placements compatible with hypothesis B imply even smaller branch lengths. The solution given by locate.fossil attaches the Messel fossils to the oldest node in the tree and implies that any resemblance between the Messel fossils and the extant testudinoids in our sample is entirely the result of plesiomorphy and homoplasy.

We recommend that empirical studies should test the adequacy of a single-rate Brownian motion model before the use of locate.fossil.

### One or two species?

In this contribution we have favoured the presentation of our results under the interpretation that *messeliana* and *kehreri* are distinct species rather than conspecific morphs. This is convenient and plausible, but we have not resolved the issue in this study. Here we will merely expand on both hypotheses and the possible interpretations of the evidence that follow from them. The results of the analyses with complete data always favoured a sister relationship between *kehreri* and *messeliana*, which is compatible with both scenarios.

Claude & Tong (2004) expressed the opinion that two morphs simply represent different ontogenetic stages. It is also possible that *messeliana* and *kehreri* represent the male and female forms of the same species. Sexual size dimorphism is common in geoemydids, in most cases with females being larger (Berry & Shine, 1980; Gibbons & Lovich, 1990; Ceballos et al., 2013). An extreme case is *Batagur dhongoka*, with a reported female-to-male mean straight-line carapace length ratio of 2.32 (Gibbons & Lovich, 1990). Fitting with that interpretation, female reproductive internal structures have been identified only in *kehreri* (Gaßner et al., 2001). Hervet (2003, p. 93) stated that male and female forms can be identified in *kehreri* by the shape of their plastra and depth of the anal notches, but she did not explain how she determined the sexes associated with each morphology, and did not make reference to specific specimens of each sex to allow us to corroborate her observations. Differences in tail lengths have been used to identify male and female specimens of the carettochelyid *Allaeochelys crassesculpta*, also from Messel, that were preserved forming mating pairs (Joyce et al., 2012). There were not enough complete or nearly complete tails among the Messel geoemydid specimens that we examined to perform similar analyses.

Conversely, it is possible that *messeliana* and *kehreri* simply represent sympatric closely related species. Sympatry of closely related species occurs in turtles. According to the Turtles of the World Checklist (Turtle Taxonomy Working Group, 2017), the following are examples of groups of closely-related geoemydid species with at least partially overlapping types of habitat that have been observed at the same localities: *Pangshura smithii*, *Pangshura tecta*, and *Pangshura sylhetensis* in Assam, India; *Rhinoclemmys melanosterna* and *Rhinoclemmys nasuta* in Chocó, Colombia; and *Heosemys annandalii* and *Heosemys grandis* in Vietnam, Thailand, and Cambodia. Furthermore, there is no reason to expect the coexistence of the two putative species to be ecologically problematic. Instead, it is reasonable to infer that there would not have been complete overlap of the niches of the two morphs, as their different body sizes would have allowed them, for instance, to specialise in different kinds of prey.

We call researchers to consider both scenarios in cases where they would plausibly influence the direct results or interpretation of their analyses.

### The phylogenetic relationships of geoemydids

We based our placement analyses and our discussion in the following section on the Pereira tree because it is the result of a recent study with what was at the time (2020) the most comprehensive sampling of species and molecular markers, and because it is broadly compatible with the results from previous studies (*e.g*. Spinks et al., 2004; Diesmos et al., 2005; Guillon et al., 2012). Coincidentally, it is also the study that found the oldest divergence times for deep geoemydid clades, presenting us with a broader range of possible placements temporally compatible with the age of the Messel fossils. Still, the assumption of a “known” fixed phylogeny in our placement analyses is purely operational, and we must highlight general outstanding issues in our current knowledge of geoemydid interrelationships.

A new phylogenetic analysis of extant turtles by Thomson, Spinks & Shaffer (2021) yielded a new hypothesis regarding the interrelationships of major geoemydid clades. In their hypothesis, *Rhinoclemmys* becomes sister to a large clade that includes *Cuora, Mauremys, Cyclemys*, and *Heosemys* rather than being sister to all other geoemydids, and *Geoemyda* becomes sister to the *Mauremys-Cuora* clade rather than to *Siebenrockiella*. These alternative relationships would significantly alter our interpretation of the phenotypic evolution of geoemydids. For instance, the presence of three carapacial keels (Claude & Tong, 2004) would become a likely synapomorphy of Geoemydidae itself. It is notable that the study of Thomson, Spinks & Shaffer (2021) made use of a mostly novel and well-sampled set of 15 nuclear loci, whereas most of the previous studies had used a high proportion of mitochondrial loci and often had patchy sampling for several loci (Spinks et al., 2004; Le & McCord, 2008; Guillon et al., 2012; Lourenço et al., 2012; Pereira et al., 2017; Colston et al., 2020). The only other phylogenetic analysis of geoemydids without mitochondrial loci is the study of Sasaki et al. (2006) based exclusively on insertion patterns of short interspersed nuclear elements (SINE), and this study also recovered a clade including, among others, *Geoemyda*, *Rhinoclemmys*, and *Mauremys* (a so-called “*Geoemyda* group”) and excluding *Siebenrockiella*, *Batagur*, and other south-east Asian geoemydids. That study, however, only sampled 21 geoemydid species and failed to resolve the relationships of *Rhinoclemmys* and *Geoemyda* within the “*Geoemyda* group”. It is likely that an important cause of the topological discrepancy of the new study of Thomson, Spinks & Shaffer (2021) is the sampling of different molecular markers that show different patterns of locus phylogenies due to incomplete lineage sorting and possibly some degree of introgression. Indeed, Sasaki et al. (2006) also recognised incomplete lineage sorting as a possible cause of conflict in SINE insertion patterns, a type of data in which homoplasy is expected to be very rare (Shedlock & Okada, 2000). Although consensus remains about the composition of geoemydid “genera” and several subclades, a detailed study using methods based on coalescent theory is needed to clarify the relationships between major geoemydid clades.

A second problem pertains to the molecular estimates of the ages of geoemydid clades. As noted before, Pereira et al. (2017) found the oldest set of ages for geoemydid clades, and other studies (Le & McCord, 2008; Lourenço et al., 2012; Joyce et al., 2013; Colston et al., 2020) found far less explicit or implied temporal overlap between the age bracket of the Messel deposit and extant geoemydid clades (Fig. 10). In particular, Colston et al. (2020) estimated far younger divergence times, with a 95% highest posterior density (95% HPD) bracket of 40.0 to 49.3 Ma for the timing of the basal split of geoemydids, and no other geoemydid clade having an age compatible with the placement of the Messel geoemydids within its divergence time 95% HPD interval. According to those age estimates, the Messel geoemydids could only be placed as a stem geoemydids or in a very basal position within the geoemydid crown. Estimates from Bayesian dating analyses can be highly sensitive to a multitude of modelling decisions, such as the choice between partitioning and coalescent models (see above), the nucleotide substitution models, the choice of clock model, the use of a tree model or fixed topology, and calibration priors. All such factors vary between the relevant studies (Le & McCord, 2008; Lourenço et al., 2012; Joyce et al., 2013; Pereira et al., 2017; Colston et al., 2020), and in none of those works there were assessments of the fit of different clock models. Furthermore, the sampling of loci (discussed above), species, and choice of divergence time calibrations are critical to every phylogenetic dating analysis. Without venturing in an assessment of the quality of the different calibration sets, it should be noted that in none of them there was more than one time-calibrated node within Geoemydidae, and that the calibration priors were typically wide. For example, the divergence between *Heosemys spinosa* and *Mauremys reevesii* set to have a flat prior distribution with a maximum of 68.5 Ma and a minimum of 5.3 Ma (Joyce et al., 2013; Pereira et al., 2017).

To complicate matters further, a recent phylogenetic analysis found several species of “*Echmatemys”* from the Eocene of North America within the crown of *Mauremys* (Vlachos, 2020; see also Vlachos, 2018). Previously, *Echmatemys* had been considered as a likely stem geoemydid or closely related to *Rhinoclemmys* (McDowell, 1964; Hirayama, 1984; Claude & Tong, 2004; Claude et al., 2012). The new result would imply much earlier origins than the current estimates for many geoemydid clades, but it should be taken with caution, as it is unclear whether the proposed relationships are well-supported. Only the strict consensus of the most parsimonious trees were reported (without bootstrap analyses), and the behaviour of analyses with the discrete character matrix that was used has not been explored in detail. Moreover, fossils of juveniles attributed to *Echmatemys* have been recently reported (Lichtig & Lucas, 2015; Lichtig, Lucas & Jasinski, 2021), bearing new morphological evidence that was not taken into account in the phylogenetic analyses. The identification of these specimens merits close revision, as they remarkably display the tricarinate condition (see below) unknown in *Echmatemys* or any other American geoemydid previously known. These specimens must be carefully compared with European fauna as fast faunal migrations likely occurred during the earliest Eocene (Claude & Tong, 2004; Lourenço et al., 2012; Smith et al., 2014).

Thus, we are left without clear means to determine which (if any) set of dates is reliable enough for fossil placement purposes. As discussed in a previous section, this affects directly the reliability of the inferences of locate.fossil, but is no problem for Cophymaru and maximum parsimony. However, it weakens the usefulness of the estimated clade ages as an external criterion for judging the plausibility of the placements estimated with the latter two methods. More generally, any difficulties that might be behind the discordance between the age estimates found in different studies are also plausible factors in our lack of success in the implementation of Bayesian inference with combined molecular and morphological data.

### The phylogenetic position of the Messel geoemydids

Following the results of the placement performance analyses, we give greater credence to the placement analyses with combined ratio and discrete data with maximum parsimony, and in this section we will refer to those results, in particular with 95% CI, unless otherwise noted. We discussed above current problems in the phylogeny of extant geoemydids, here we will only concentrate on the relationships of the Pereira tree, for simplicity.

**Figure 11:**
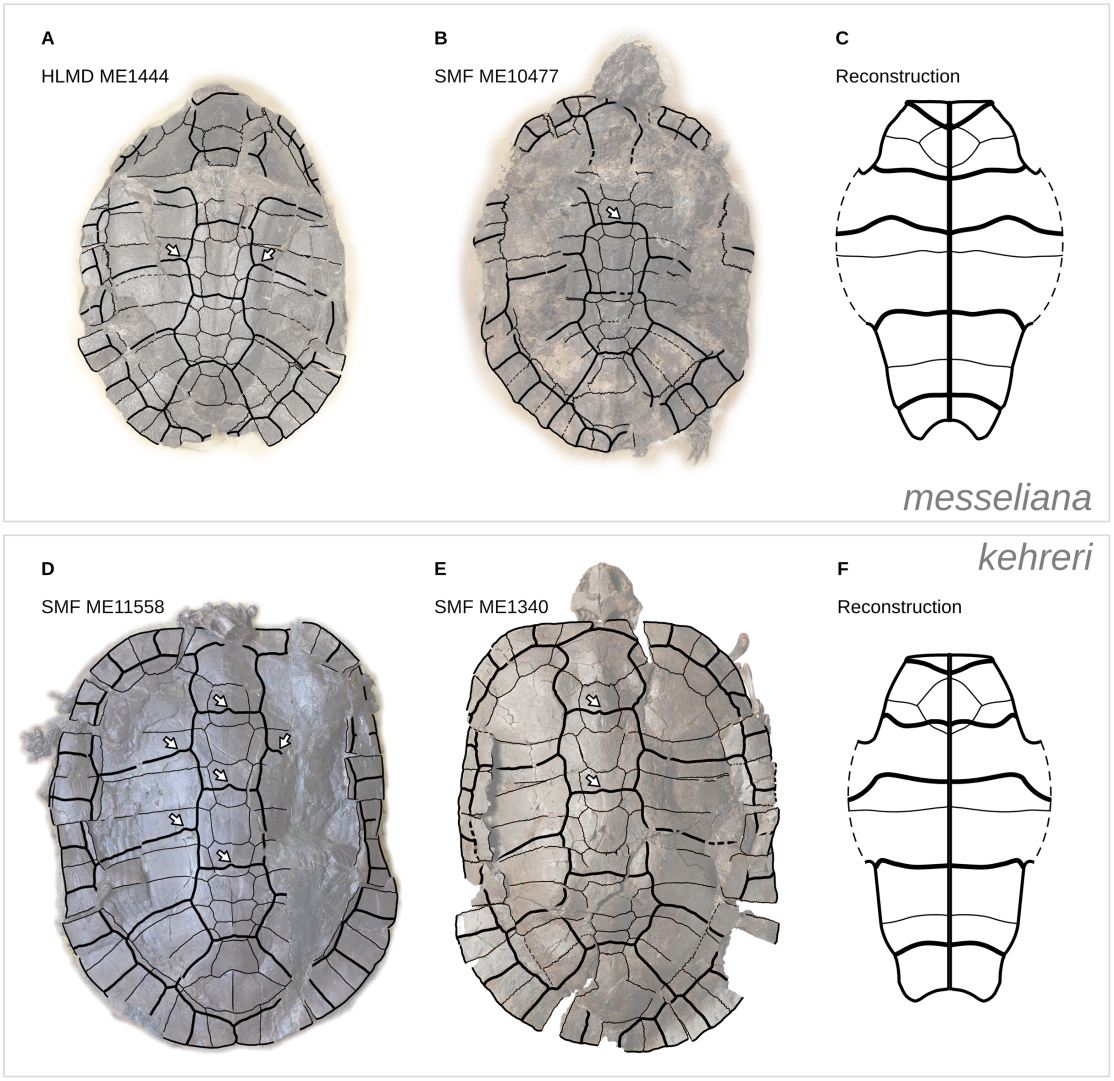
Representative carapaces and reconstructions of the plastra of *messeliana* (A–C) and *kehreri* (D–F). The plastron reconstructions are based on measurements of the fossils. White arrows indicate sulcus inflections that we interpret to mark the current or former presence of carapacial keels (see *Mauremys annamensis* and *Geoemyda* in Fig. 12). HLMD ME1444 is the holotype of *messeliana*. Not to scale.

In all the analyses except locate.fossil, the Messel geoemydids are firmly placed in the clade of tricarinate (or “three-keeled”) geoemydids. This clade is the sister to *Rhinoclemmys*, and includes all the other extant geoemydid species. The tricarinate condition refers to the occurrence of two lateral carapacial keels (character D1, state 2; Fig. 11) that do not occur in *Rhinoclemmys* and other testudinoids (Claude & Tong, 2004; Joyce & Bell, 2004; Garbin, Ascarrunz & Joyce, 2018). Placements outside the tricarinate geoemydid clade received negligible bootstrap support in the combined evidence analysis (Fig. 7) and the parsimony and Bayesian analyses with log-ratio data alone (Fig. 8A) or discrete data alone (Fig. 8B).

**Figure 12:**
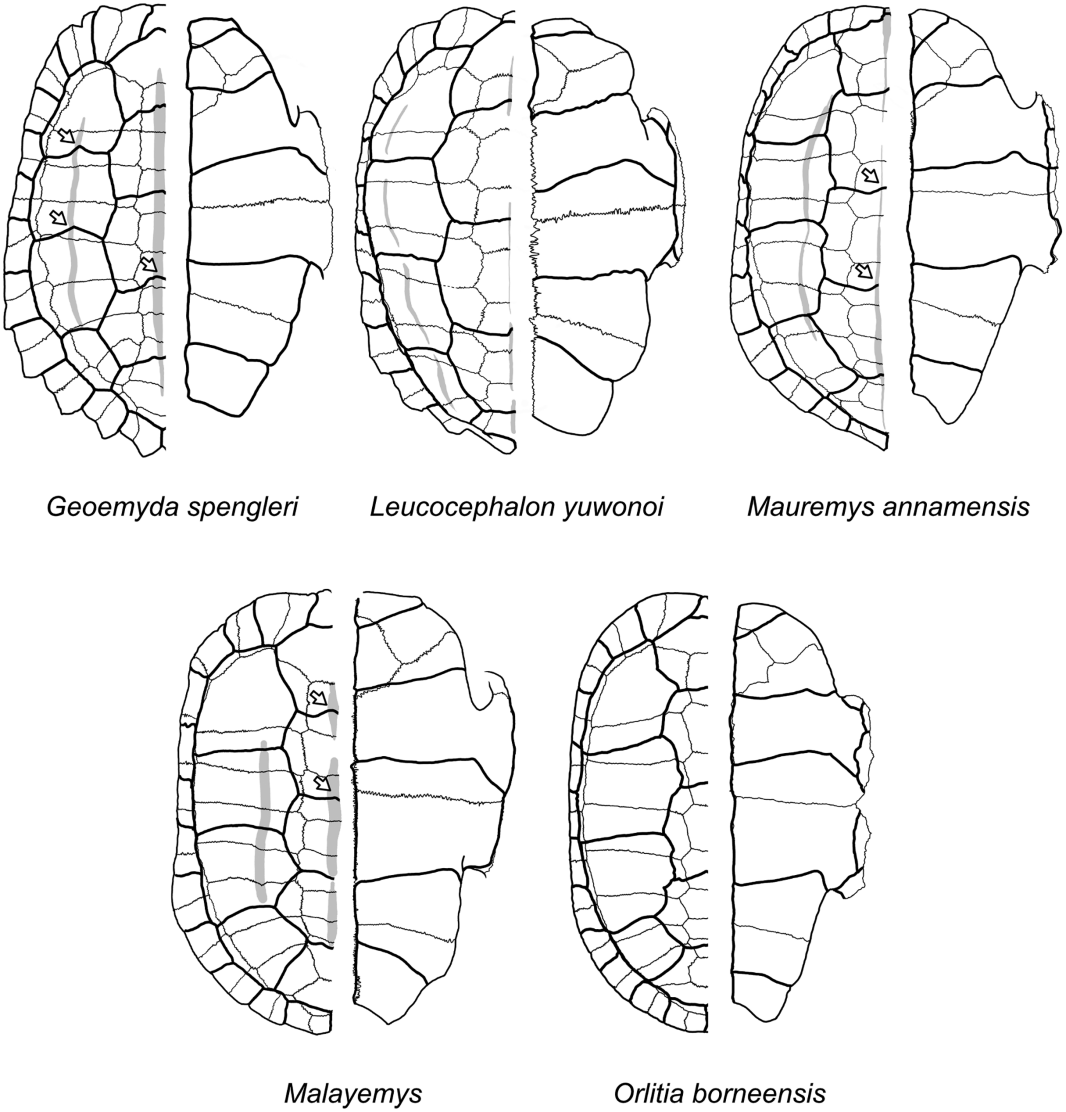
Diagrams of carapaces and plastra of extant geoemydids. Carapacial keels are indicated with light grey. Not to scale. White arrows mark sulcus inflections that are coincidental with carapacial keels. *Geoemyda spengleri* is based on PCHP 12207; *Leucocephalon yuwonoi* is based on YPMHERR 12109, YPMHERR 17221, and MTD 40171 from the Museum für Tierkunde in Dresden, Germany; *Mauremys annamensis* is based on PCHP 4071; *Malayemys* is based on PCHP 3446; and *Orlitia borneensis* is based on PCHP 3366. Note that sutures in the pygal region of *Leucocephalon yuhonoi* are not depicted only because they were obscured in our reference photographs.

We give less credence to the placement of *messeliana* as sister to *Batagur dhongoka* and *kehreri* as sister to *Leucocephalon yuwonoi* because they are strongly supported only when the discrete data are excluded. The placement of *kehreri* as sister to *Leucocephalon yuwonoi* is particularly dubious because *Leucocephalon yuwonoi* itself is a problematic species to place in the reference tree in the placement performance analyses. Even with combined discrete and ratio data and the optimal cost scheme, its scaled placement error is 0.5. Like the Messel geoemydids (Fig. 11) and *Geoemyda spengleri* (Fig. 12), *Leucocephalon yuwonoi* has short and wide gulars that reach about the anterior tip of the entoplastron (RP1 = 0.16, RP8 = 2.68, RP12 = 1.04; for ease of interpretation, all the ratio character values that we give in this section are raw means, not log-transformed). However, the overall shape of the epiplastra and the gulars of *Leucocephalon yuwonoi* is also markedly different (Fig. 12): the gulo-humeral sulcus is more horizontal and marks a deep waist in the epiplastron. Furthermore, we do not see other distinctive putative synapomorphies for this placement in other characters. For instance, *Leucocephalon yuwonoi* does not have the strong plastral buttresses shared by *kehreri, Malayemys, Orlitia*, and *Batagur baska*. And, although we did not focus our attention on the skulls of the Messel geoemydids, it is clear that their gross morphology is rather typical of geoemydids and does not show the peculiar features noted in the diagnoses of *Leucocephalon yuwonoi* (McCord et al., 1995, 2000). The sufficiently preserved skulls of *kehreri* (HMLD-ME 8877, HLMD-ME 15033, SMF-ME 1340) and *messeliana* (SMF-ME 1210) display a robust temporal bar (“postorbital bar” in McCord et al., 1995, 2000), which *Leucocephalon* lacks entirely due to loss or great reduction of the quadratojugal (McCord et al., 1995, 2000; our observation of YPMHERR 12109). We have also observed absent or slender temporal bars in collection specimens of the species that form the sister clade to *Leucocephalon yuwonoi*: *Cyclemys dentata, Notochelys platynota, Heosemys grandis*, and *Heosemys spinosa*. Thus, placements sister to *Leucocephalon* would imply homoplastic reduction of the temporal bar or a reversion to the primitive condition. Another skull feature that *Leucocephalon yuwonoi* does not have in common with the Messel geoemydids is the presence of a medial anterior contact of the maxillae. In skulls of *kehreri* (HLMD-ME 8877, HLMD-ME 15033, SMF-ME 1340) the premaxillae prevent the anterior contact of the maxillae, and the bony “beak” at the anterior end of *kehreri* skulls (HLMD-ME 8877, HLMD-ME 15033, SMF-ME 1340) has the typical notch found in most geoemydids rather than the “hook” seen in *Leucocephalon yuwonoi*.

The most parsimonious placement with combined discrete and log-ratio data depends on the use of 95% confidence intervals of the means (hypothesis B, cherry sister to *Orlitia borneensis*) or just the means of the log-ratio characters (hypothesis C, cherry sister to *Geoemyda spengleri*), but with either treatment the bootstrap support is concentrated in results forming cherries in the branches corresponding to hypothesis B or in a sister relationship to *Geoemyda spengleri* (Fig. 7).

In Fig. 9 we show that characters RP1, RP8, and RP12 more strongly favour hypothesis C over the placements of hypothesis A or B. All the three characters pertain to the gular scutes. RP1 represents the length of the intergular sulcus relative to the total length of the midline sulci of the anterior plastral lobe. RP8 is the ratio between the anterior width of the gular to the intergular sulcus. R12 is the ratio between the interepiplastral suture and the intergular sulcus, which represents the midline position of the gulo-humeral sulcus. Values of RP12 smaller than 1 normally imply that the gulo-humeral sulcus overlays the entoplastron. Very low values of RP1, high values of RP8, and values of RP12 close to 1 (*i.e*., short and wide gulars that reach about the anterior tip of the entoplastron) are found in *kehreri* (RP1 = 0.14, RP8 = 2.33, RP12 = 1.05) and *messeliana* (RP1 = 0.17, RP8 = 1.76, RP12 = 0.87), and also *Leucocephalon yuwonoi* (RP1 = 0.16, RP8 = 2.68, RP12 = 1.04), *Geoemyda spengleri* (RP1 = 0.17, RP8 = 1.91, RP12 = 0.91). Although it is possible, for instance, for a gular scute to be short, narrow, and still reach the entoplastron, it is likely that there is some significant correlation between characters RP1, RP8, and RP12, and we may thus have given too much weight to the features of the gulars. And notably, removing either of them considerably shifts the amounts of homoplasy in favour of hypotheses A and B (Fig. 9). An independent assessment of the three characters might be necessary to rectify a possible overweighting error. Other characters that are strongly against the hypothesis C compared to hypothesis A and hypothesis B are the position of the lateral keels (D2), the serration of the posterior peripherals (D19) and the length of the interabdominal sulcus (RP3). A particularly problematic aspect of hypothesis C is that we also found *Geoemyda* to be difficult to place in the tree in the placement performance analyses (scaled nodal error = 0.55, with either the means or confidence intervals of log-ratio characters).

Hypothesis B received 49% of bootstrap support combining all the possible placements within the *Orlitia*-*Malayemys* clade or its stem (Fig. 7). As noted by Claude & Tong, this placement is supported by the position of the lateral keels closer to the neural series than midway between the neural series and the peripheral series (character D2, state 1; Fig. 9). *Orlitia borneensis* also shows poor placement performance when 95% CIs of the means of the log-ratio characters are used (scaled placement error = 0.75), probably due to the high variance observed in some log-ratio characters for this species. When point estimates of the means are used, the placement performance of *Orlitia borneensis* is much better (scaled placement error = 0.25).

Hypothesis A received little bootstrap support in the analysis of combined data, with a total of 2% or 3% of the bootstrap placements (Fig. 7). The most parsimonious placement consistent with hypothesis A was as a cherry sister to *Mauremys annamensis*. This species is endemic to Vietnam (Turtle Taxonomy Working Group, 2017), and was not included in the phylogenetic analyses conducted by Hervet (2003). The placement sister *Mauremys annamensis* is supported by the same position of the lateral keels as seen in *Orlitia* and *Malayemys* (character D2, state 1). In this feature *Mauremys annamensis* differs from the other extant *Mauremys* that display the more widespread condition with the lateral keels farther away from the neural series, but lateral keels close to the neural series are also found in *Mauremys thanhinensis* from the late Eocene-early Oligocene of the Krabi Basin in Thailand, and could represent the ancestral condition of *Mauremys* (Claude, Suteethorn & Tong, 2007). With point estimates of the means of the log-ratio characters and light implied weights, the only character that strongly supports hypothesis A over both the hypothesis C and hypothesis B is RC1, which refers to the length versus width of the second marginal. Indeed, the similarity with the Messel geoemydids in the anterior marginals is clearly visible (Fig. 11, Fig. 12). When 95% CIs of the means of the log-ratio characters and equal weights are used, various ratio characters favour hypothesis A over hypothesis B by slight amounts, but they are offset by vast score differences in RP1, RP9 (size of the xiphiplastron) and the discrete characters D12, D52, and D53.

Of particular interest is also the classical character RP6, which corresponds to the position of the humero-pectoral sulcus relative to the entoplastron. Greater values of RP6 reflect more posterior positions of the humero-pectoral sulcus, and values smaller than 1.0 indicate that the sulcus is located anterior enough to overlay the entoplastron. This character has been used in discrete forms in many previous studies (Das, 1997; Hervet, 2003; Claude & Tong, 2004; Joyce & Bell, 2004; Takahashi et al., 2013; Garbin, Ascarrunz & Joyce, 2018), and was originally used for the differential diagnosis of *messeliana* (sulcus posterior to entoplastron, RP6 = 1.18) and *kehreri* (sulcus overlying entoplastron; mean RP6 = 0.89, but SMF ME3774 has an RP6 = 1.03, with the sulcus slightly posterior to the entoplastron) (Staesche, 1928; Hervet, 2003, 2004) (Fig. 11). Claude & Tong (2004) considered that the humero-pectoral sulcus well posterior to the entoplastron was diagnostic for *Palaeoemys*, but their synonymisation of *kehreri* and *messeliana* with *Palaeoemys messeliana* implies that their diagnosis must be amended to account for the more anterior position of the sulcus observed in the *kehreri*. *Geoemyda spengleri* has an RP6 = 0.76, *Mauremys annamensis* has an RP6 = 0.853, *Orlitia borneensis* has an RP6 = 1.09, and *Malayemys* has an RP6 = 0.94 (Fig. 12). Character RP6 is a good illustration of the utility of continuous characters. The posterior suture of the entoplastron represents a convenient point of reference for defining discrete character states, but it is an arbitrary threshold that does not accurately represent the magnitude of the differences between the phenotypes and would have introduced noise in the analyses in previous studies.

## Conclusions

We do not give a definite answer to the specific placement of the Messel geoemydids, but we believe that this study demonstrates the empirical informativeness of continuous characters in palaeontological placement analysis (Parins-Fukuchi, 2018a; Ascarrunz, Claude & Joyce, 2019). This kind of analysis is in a sense less ambitious than the inference of entire trees based on continuous characters alone, a debate that has been recently rekindled (Parins-Fukuchi, 2018b; Varón-González, Whelan & Klingenberg, 2020), but which we do not touch in the present contribution. Among the methods evaluated, a model of Brownian motion without a fixed rate of evolution and free branch lengths showed again an edge over maximum parsimony. The integration of Brownian models for continuous characters with traditional discrete characters remains an alluring project despite our current failure to implement it.

Although we found in *Mauremys annamensis* a new potential link between *Mauremys* and the Messel geoemydids, hypothesis A is less parsimonious than hypothesis B and hypothesis C, and it received very low bootstrap support, recasting serious doubts over the idea of the “*Palaeochelys sensu* lato–*Mauremys*” complex. When all the characters are included, either hypothesis B or the placement sister to *Geoemyda* are the optimal solution depending on the treatment of intraspecific variation of the log-ratio characters, but both receive at least modest bootstrap support. Other characters not included in the analyses are consistent with hypothesis B (robust temporal bar, thick plastral buttresses). However, the more medial position of the lateral carinae is also found in other clades (*Mauremys*, and one geoemydid from the Eocene of North America), which suggests that this key character identified by Claude & Tong (2004) in support hypothesis B could be more prone to homoplasy than originally thought. The new hypothesis of the close relationship between the Messel geoemydids and *Geoemyda* merits more attention and scrutiny because of the possible influence of character correlations and homoplasy (as reflected in the difficulty to place *Geoemyda* in the placement performance analyses), and because *Geoemyda* finds itself at the centre of a possible major revision of the relationships between major geoemydid clades (Thomson, Spinks & Shaffer, 2021). Alternative hypotheses about the topology and timing of the deep relationships between extant geoemydids could also have a strong effect on the inferred placement of the Messel geoemydids. The early age of the fossils and primitive traits of the skull could be consistent with the Messel geoemydids belonging to an extinct lineage in a more basal position. We encourage other researchers to consider account these uncertainties in their future work, and at least take into account hypothesis C and the placements of hypothesis B as plausible resolutions.

A nomenclatural revision of *kehreri* and *messeliana* remains outstanding. Even if the “*Palaeochelys sensu* lato–*Mauremys*” group is rejected, it is necessary to study with explicit phylogenetic methods a wider sample of the material studied by Hervet to assess the monophyly of the genera that she erected. Likewise, the more ample concept of *Palaeoemys* of Claude & Tong (2004) remains to be assessed with methods similar to the ones used in the present study (Mongiardino Koch, Garwood & Parry, 2021).

## Supplemental Information

10.7717/peerj.11805/supp-1 Supplemental Information 1 Appendix describing the model and results of the RevBayes analyses. The analyses described herein were unsuccessful and their results are not reported in the paper.

10.7717/peerj.11805/supp-2 Supplemental Information 2 Data files and scripts for reproducing the main analyses. The raw landmark data, the raw segment measurements, and the matrices with combined discrete and continuous characters together with the files necessary for reproducing the main parsimony analyses. Consult the file README.md contained therein for a description of the contents and instructions for reproducing the analyses.

## Additional Information and Declarations

### Data Availability

The following information was supplied regarding data availability: The continuous data (landmark coordinates and segment measurements), together with the discrete character data and additional files to reproduce the main analyses, are available in the Supplemental File.
