# Supplementary figures and images for: The phylogenetic relationships of geoemydid turtles from the Eocene Messel Pit Quarry: a first assessment using methods for continuous and discrete characters
Source: PeerJ. 2021 Aug 5;9:e11805. doi: 10.7717/peerj.11805 (PMC8349520; doi:10.7717/peerj.11805)

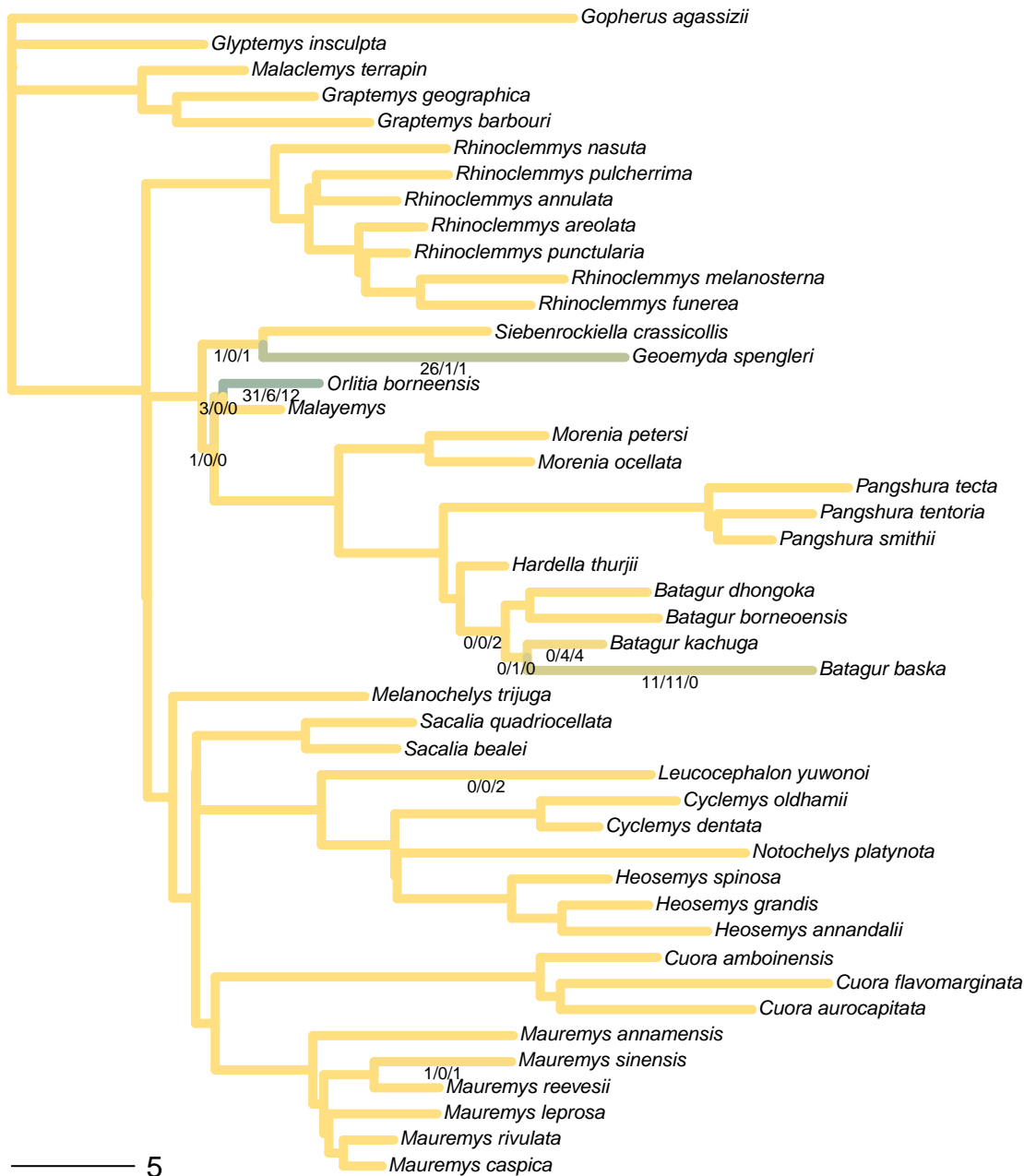

Supplement: Supplemental Information 2 — The raw landmark data, the raw segment measurements, and the matrices with combined discrete and continuous characters together with the files necessary for reproducing the main parsimony analyses. Consult the file README.md contained therein for a description of the contents and instructions for reproducing the analyses. [file peerj-09-11805-s002.zip › data_and_scripts/main_analyses/3_parsimonyci/place/output/combined_zscale_bootplot.pdf]

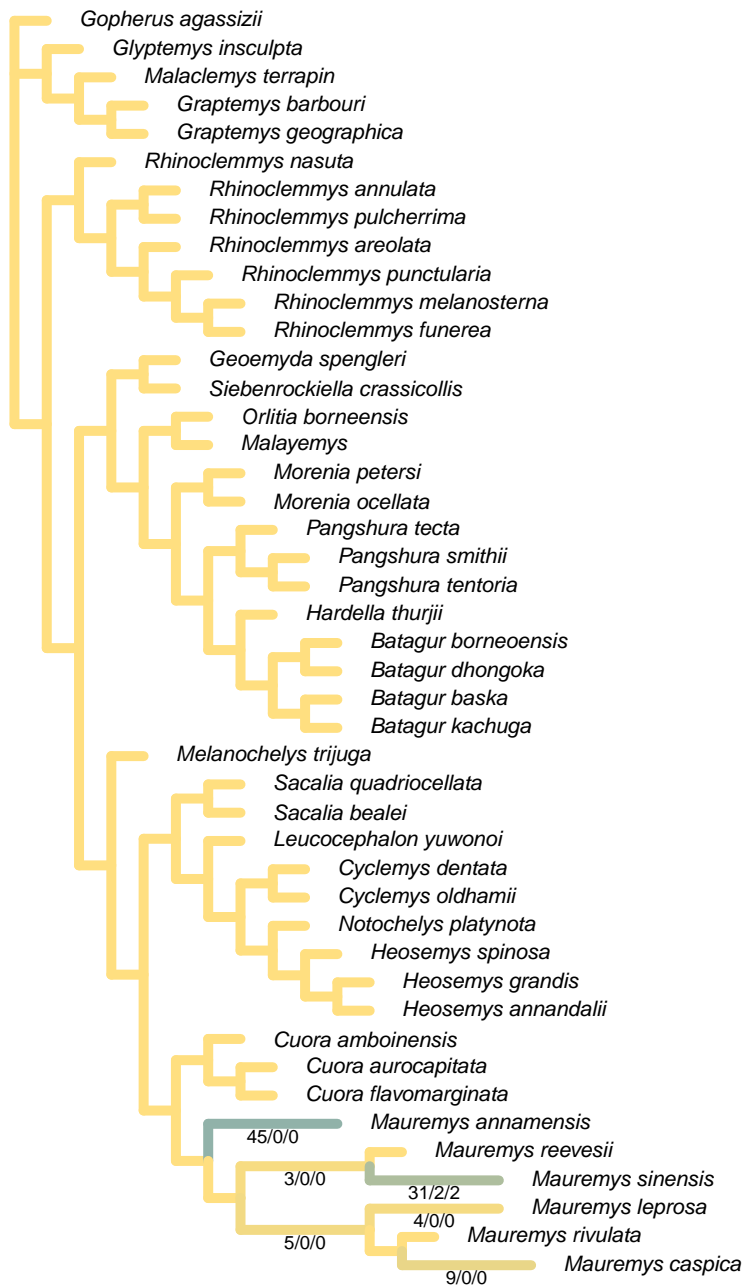

Supplement: Supplemental Information 2 — The raw landmark data, the raw segment measurements, and the matrices with combined discrete and continuous characters together with the files necessary for reproducing the main parsimony analyses. Consult the file README.md contained therein for a description of the contents and instructions for reproducing the analyses. [file peerj-09-11805-s002.zip › data_and_scripts/main_analyses/3_parsimonyci/place/output/hypothesis_a_bootplot.pdf]

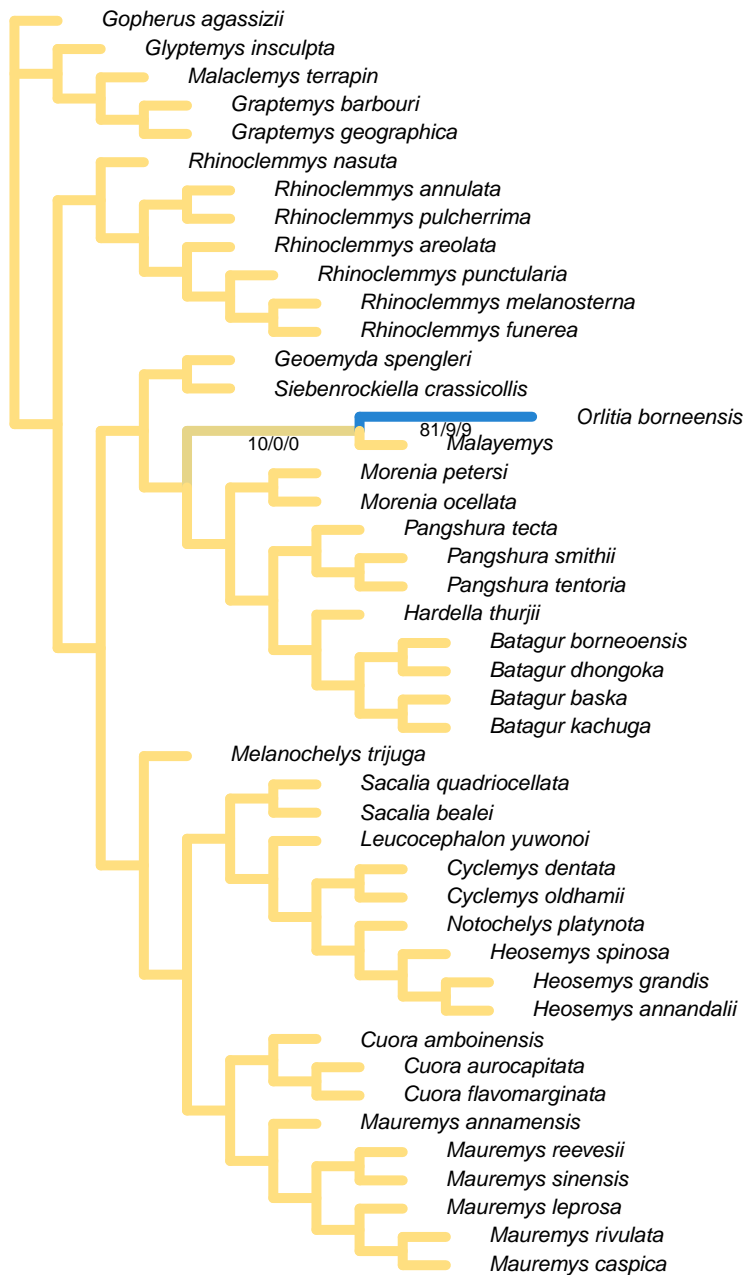

Supplement: Supplemental Information 2 — The raw landmark data, the raw segment measurements, and the matrices with combined discrete and continuous characters together with the files necessary for reproducing the main parsimony analyses. Consult the file README.md contained therein for a description of the contents and instructions for reproducing the analyses. [file peerj-09-11805-s002.zip › data_and_scripts/main_analyses/3_parsimonyci/place/output/hypothesis_b_bootplot.pdf]

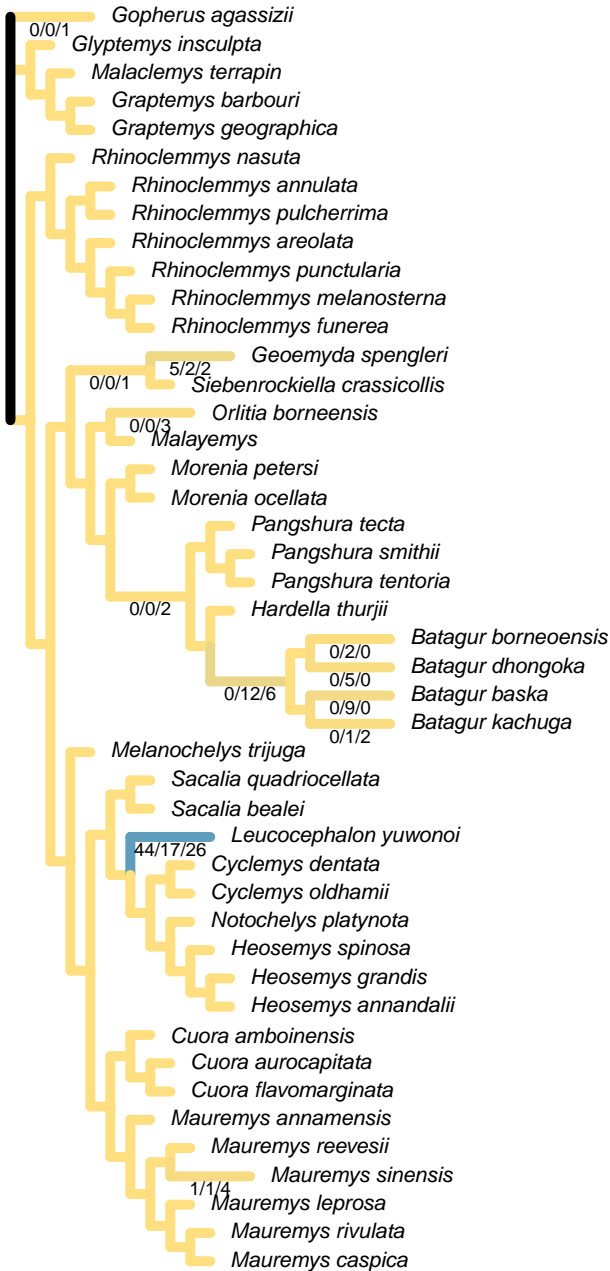

Supplement: Supplemental Information 2 — The raw landmark data, the raw segment measurements, and the matrices with combined discrete and continuous characters together with the files necessary for reproducing the main parsimony analyses. Consult the file README.md contained therein for a description of the contents and instructions for reproducing the analyses. [file peerj-09-11805-s002.zip › data_and_scripts/main_analyses/3_parsimonyci/place/output/ratios_bootplot.pdf]

$\Delta$ Score

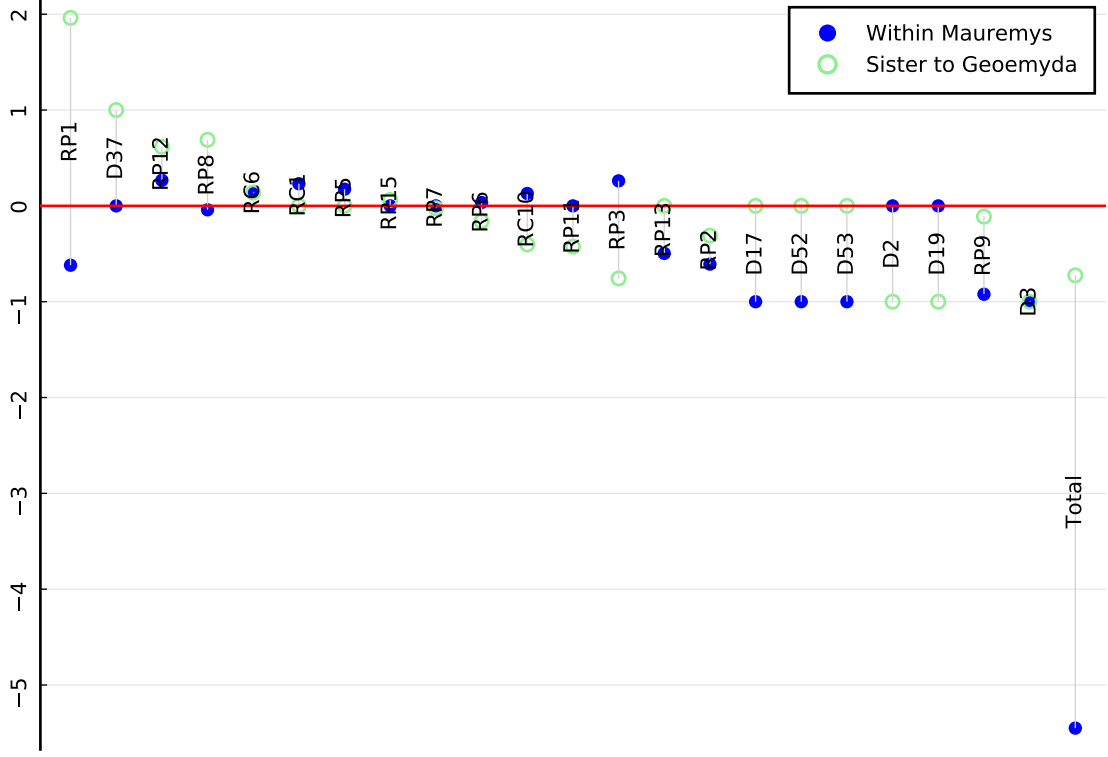

Supplement: Supplemental Information 2 — The raw landmark data, the raw segment measurements, and the matrices with combined discrete and continuous characters together with the files necessary for reproducing the main parsimony analyses. Consult the file README.md contained therein for a description of the contents and instructions for reproducing the analyses. [file peerj-09-11805-s002.zip › data_and_scripts/main_analyses/3_parsimonyci/place/output/scores_hypotheses.pdf]

$\Delta$ Score

-1

0

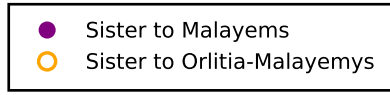

RP3

RP12

RP5

RP15

RP2

RP9

RP4

RP11

RP14

RP10

Total

Supplement: Supplemental Information 2 — The raw landmark data, the raw segment measurements, and the matrices with combined discrete and continuous characters together with the files necessary for reproducing the main parsimony analyses. Consult the file README.md contained therein for a description of the contents and instructions for reproducing the analyses. [file peerj-09-11805-s002.zip › data_and_scripts/main_analyses/3_parsimonyci/place/output/scores_malayemys.pdf]
