# Supplementary material for: The phylogenetic relationships of geoemydid turtles from the Eocene Messel Pit Quarry: a first assessment using methods for continuous and discrete characters
Source: PeerJ. 2021 Aug 5;9:e11805. doi: 10.7717/peerj.11805 (PMC8349520; doi:10.7717/peerj.11805)
Supplement: Supplementary file 178 [file peerj-09-11805-s001.odt]

Supplemental information 1 (Appendix): Bayesian analysis of combined data

We implemented a complex model for the joint Bayesian analysis of log-ratio characters and discrete characters. Models like this are not built-in in standard phylogenetic inference programs. Instead, we used the software RevBayes (Höhna et al., 2016), in which the user is provided with a special-purpose programming language for specifying custom probabilistic graphical models of phylogenetic data for Bayesian analysis with Markov Chain Monte Carlo (MCMC). Graphical probabilistic models are representations of statistical models as directed graphs (specifically directed acyclic graphs in RevBayes). The nodes of the graph represent model variables and directed edges (arrows) represent the conditional dependencies between them. This graphical system naturally provides simple and exact visual representations of complex models. See Höhna et al. (2014) for a detailed description of the theory and visual conventions and Fig. 1 for a very brief description of the basic visual elements. In the following, we will use the graphical visualisations to present our model. All the other implementation details (e.g. MCMC moves) are described as code in the RevBayes language in scripts in the electronic supplemental information (S2), so we also show the equivalences between the symbols in the figures and the variable names used in the scripts. Stochastic are also shown with their respective distribution names in the RevBayes language.

For convenience of exposition and implementation, we divided the model in several interconnected modules. Note that sometimes we use the same symbol to denote different variables in different modules. Also, many of the variables used here are vectors. We use the square bracket notation where x[i] represents the ith element of vector x.

The entire model is put together in a script called “master.Rev”. An overview of the entire model is given in Fig. 2. The modular design also allowed us to easily implement variations of the model. We describe two variations of the model: one with uncorrelated exponential clocks and another with uncorrelated lognormal clocks. Several other aspects of the model are specified in settings files written for each analysis. The files “uce_1.Rev” and “ucln_1.Rev” are examples of setting files with the uncorrelated exponential and the uncorrelated lognormal clock models, respectively.

Our analyses did not succeed in yielding biologically interpretable results. We performed four MCMC runs of our model variations, which we cut stopped as they began to show aberrant behaviour. This problem is shown in the final section of the annex.

Figure 1. Visual elements of a graphical probabilistic model. Square constant nodes (A) are parameters with constant values. Stochastic nodes (B) are represented by solid circles and correspond to random variables with explicit probability distributions whose parameters may be represented by other nodes. Deterministic nodes appear as circles with dashed outlines (C) and represent functions that take inputs from other nodes. Shaded solid circles are clamped stochastic nodes and correspond to random variables whose values have been fixed (“clamped”) due to the observation of data. Plates are represented as dashed rectangles, and they represent replication over a set of variables. The replication is specified by an iterator variable indicated in a corner of the plate. We introduce an additional convention for modular graphs: Yellow circles represent stochastic variables from external modules, with the modules themselves appearing as rectangles with rounded corners. 

Figure 2. Dependencies between the variables of all the modules of the model.

# Tree module

Figure 3. Visual representation of the script “tree.Rev”.

| Symbol | Meaning | Variable name | Distribution or value |
| --- | --- | --- | --- |
| TexMaths12§display§\Psi§svg§600§FALSE§ | Topologically constrained time tree | Psi | dnConstrainedTopology |
| TexMaths12§display§\mathcal{T}§svg§600§FALSE§ | Birth-death process | treedist | dnBDP |
| TexMaths12§display§\Psi_0§svg§600§FALSE§ | Topological constraint tree | backbone | Pereira tree |
| TexMaths12§display§\phi§svg§600§FALSE§ | Time of origin of the birth-death process | age_root | dnNormal |
| TexMaths12§display§\lambda§svg§600§FALSE§ | Speciation rate | speciation_rate | dnExponential |
| TexMaths12§display§\mu§svg§600§FALSE§ | Extinction rate | extinction_rate | dnExponential |

The prior distribution for the trees is a topologically constrained birth-death process, where speciation and extinction rates are given diffuse exponential priors with a mean parameter of 0.1 speciation and extinction events per Ma. The diversification parameters are not of interest in this study, and our fossil sampling is inadequate for estimating them reliably. Therefore, we fixed the extant sampling parameter ρ to 1.0. Based on the prior used by Joyce et al. (2013) for the origin of Testudinoidea, the prior for the origin of the birth-death process (the “age of the root”) is a normal distribution with a mean of 97.65 Ma and a standard deviation of 15 Ma, truncated at 145 Ma and 50.3 Ma. That prior also places a high probability mass around the 92.1 and 95 Ma mean age estimates for the origin of Testudinoidea of Pereira at al. (2017) and Lourenço et al. (2012), and less so around the 85.83 Ma estimate of Joyce et al. (2013). The topological constraint is the Pereira tree.

# Clock modules

Clock models describe the variation in the rates of evolution across the branches of the tree. The modules that we used here contain two separate clocks with the same structure for the morphological data and the molecular data. The clock modules that we describe here correspond to uncorrelated exponential clocks and uncorrelated lognormal clocks. We also experimented with an autocorrelated lognormal clock model (Thorne & Kishino, 2002), not shown here. 

## Uncorrelated exponential clocks

Figure 4. Visual representation of the script “uce_rates.Rev”.

| Symbol | Meaning | Variable name | Distribution or value |
| --- | --- | --- | --- |
| TexMaths12§display§\mu_{r_{M}}§svg§600§FALSE§ | Mean morphology branch rate | uce_rates_morphology_mean | dnExponential |
| TexMaths12§display§r_M[i]§svg§600§FALSE§ | Morphology rate of branch TexMaths12§display§i§svg§600§FALSE§ | br_rates_morphology[i] | dnExponential |
| TexMaths12§display§\mu_{r_{G}}§svg§600§FALSE§ | Mean molecular branch rate | uce_rates_dna_mean | dnExponential |
| TexMaths12§display§r_G[i]§svg§600§FALSE§ | Molecular rate of branch TexMaths12§display§i§svg§600§FALSE§ | br_rates_dna[i] | dnExponential |
| TexMaths12§display§n_b§svg§600§FALSE§ | Number of branches in the tree | n_branches | 92 |

Each of the branches of the tree is assigned a rate of evolution independently drawn from an exponential distribution with mean parameter drawn from another exponential distribution with a mean of 0.1. Notice that there are two separate clocks for the rate of evolution of morphology and molecular data.

## Uncorrelated lognormal clocks

Figure 5. Visual representation of the script “ucln_rates.Rev”.

| Symbol | Meaning | Variable name | Distribution or value |
| --- | --- | --- | --- |
| TexMaths12§display§\bar{r}_{M}§svg§600§FALSE§ | Mean morphology branch rate | ucln_morphology_mean | 0.0324752 |
| TexMaths12§display§\mu_M§svg§600§FALSE§ | Location parameter | ucln_morphology_mu |  |
| TexMaths12§display§\sigma_M§svg§600§FALSE§ | Standard deviation of morphology branch rate | ucln_morphology_sigma | dnExponential |
| TexMaths12§display§\sigma^2_M§svg§600§FALSE§ | Variance of morphology branch rate | ucln_morphology_var |  |
| TexMaths12§display§r_{M}[i]§svg§600§FALSE§ | Morphology rate of branch TexMaths12§display§i§svg§600§FALSE§ | br_rates_morphology[i] | dnLognormal |
| TexMaths12§display§\bar{r}_{G}§svg§600§FALSE§ | Mean molecular branch rate | ucln_dna_mean | 0.002028123 |
| TexMaths12§display§\mu_G§svg§600§FALSE§ | Location parameter | ucln_dna_mu |  |
| TexMaths12§display§\sigma_G§svg§600§FALSE§ | Standard deviation of molecular branch rate | ucln_dna_sigma | dnExponential |
| TexMaths12§display§\sigma^2_G§svg§600§FALSE§ | Variance of molecular branch rate | ucln_dna_var |  |
| TexMaths12§display§r_{G}[i]§svg§600§FALSE§ | molecular rate of branch TexMaths12§display§i§svg§600§FALSE§ | br_rates_dna[i] | dnLognormal |
| TexMaths12§display§n_b§svg§600§FALSE§ | Number of branches in the tree | n_branches | 92 |

The structure of this submodel is similar to its exponential counterpart (see previous section), but the branch rates are drawn from a lognormal distribution. We calculated expected values (means) for that lognormal distribution by dividing mean cophenetic distances of the molecular and log-ratio character partitions by the twice the mean of the prior for the age of the root.

# Age calibrations module

Figure 6. Visual representation of the script “calibrations.Rev”. The plate was not implemented as a real loop.

| Symbol | Meaning | Variable name | Distribution or value |
| --- | --- | --- | --- |
| TexMaths12§display§t_{max}[i]§svg§600§FALSE§ | Maximum age of clade TexMaths12§display§i§svg§600§FALSE§ | Prefixed with “maxage_” | See calibration table |
| TexMaths12§display§t_{min}[i]§svg§600§FALSE§ | Minimum age of clade TexMaths12§display§i§svg§600§FALSE§ | Prefixed with “minage_” | See calibration table |
| TexMaths12§display§w_t[i]§svg§600§FALSE§ | Half of the calibration range | Prefixed with “widthage_” |  |
| TexMaths12§display§\psi[i]§svg§600§FALSE§ | Clade | Prefixed with “clade_” | See calibration table |
| TexMaths12§display§t[i]§svg§600§FALSE§ | Age | Prefixed with “age_” |  |
| TexMaths12§display§T[i]§svg§600§FALSE§ | “Observed” age | Prefixed with “obs_age” | dnUniform |
| TexMaths12§display§K§svg§600§FALSE§ | Number of calibrations | (No variable) | 4 |

RevBayes does not allow the application of priors on node age in addition to the tree-wide prior from the birth-death process. Instead, internal node calibrations must be done in a more roundabout way. The ages of the fossils used for the calibrations are treated like random variables with observed data. We follow the procedure described in a RevBayes tutorial (Höhna & Heath, 2016) where auxiliary variables are defined thus:

	TexMaths12§display§w_t = (t_{max} - t_{min}) / 2
§svg§600§FALSE§

	TexMaths12§display§T \sim Uniform(t - w_t, t + w_t)§svg§600§FALSE§

The following node calibrations were adapted from Joyce et al. (2013) , and expanded with the findings of Cuora pitheca (Naksri et al., 2013) and Pangshura tatrotia (Joyce & Lyson, 2010).

| Clade | TexMaths12§display§t_{max}§svg§600§FALSE§ (Ma) | TexMaths12§display§t_{min}§svg§600§FALSE§ (Ma) |
| --- | --- | --- |
| Cuora | 66 | 9 |
| Emydidae | 100.5 | 32 |
| Pangshura | 100.5 | 2.59 |
| Testuguria | 100.5 | 50.3 |

# Morphological substitution module

Figure 7. Visual representation of the script “morphology_susbtitution.Rev”.

| Symbol | Meaning | Variable name | Distribution or value |
| --- | --- | --- | --- |
| TexMaths12§display§\alpha§svg§600§FALSE§ | Shape parameter of ASV gamma distribution | alpha_discrete_morpho | dnUniform |
| TexMaths12§display§\mu_k§svg§600§FALSE§ | Rate-scaled ASV gamma distribution for TexMaths12§display§k§svg§600§FALSE§ character states | mu2, mu3, mu4, mu5 |  |
| TexMaths12§display§m_{Mkv}§svg§600§FALSE§ | Overall rate scaler | m_Mkv | dnExponential |
| TexMaths12§display§Q_x§svg§600§FALSE§ | JC69 instantaneous transition matrix for partition TexMaths12§display§x§svg§600§FALSE§ | Q2, Q3o, Q3u, Q4o, Q5o |  |
| TexMaths12§display§S_x§svg§600§FALSE§ | Phylogenetic continuous-time Markov chain of partition TexMaths12§display§x§svg§600§FALSE§ | zk2, zk3o, zk3u, zk4, zk5 | dnPhyloCTMC (clamped to discrete character partitions) |

We partitioned the discrete characters by their number of states (2 to 5) and whether they were ordered or unordered (o or u), as combinations of those two properties require different instantaneous transition rate matrices (Q matrices). Similar partitioning schemes (Gavryushkina et al., 2017; King et al., 2017) are becoming increasingly common and are now created by default by BEAUti for BEAST 2.5 (Bouckaert et al., 2019). The Q matrices assigned to the partitions of unordered characters have a single rate for all types of transition, following the Mk model of Lewis (2001). Partitions of ordered characters were assigned Q matrices with a single transition rate for transitions between adjacent states (e.g. 0 to 1, 2 to 3), and forbidden transitions between non-adjacent states (e.g. 0 to 2, 1 to 3). There is a single base transition rate free parameter, which is multiplied by a factor of k/2 where k is the number of states of the partition (King et al., 2017). All partitions share an among-site rate variation parameter corresponding to a 4-category discretised Gamma distribution and an ascertainment bias correction for variable characters only (Lewis, 2001). The shape parameter of the Gamma distribution was assigned a uniform prior between 0 and 103. Continuous log-ratio characters and discrete characters share the same set of branch rates, but these two kinds of characters have different units of change. We address this difference by multiplying the substitution rates of the discrete characters by a scaling parameter TexMaths12§display§m_{Mkv}§svg§600§FALSE§.

# Molecular substitution module

Figure 8. Visual representation of the script “dna_substitution.Rev”.

| Symbol | Meaning | Variable name | Distribution or value |
| --- | --- | --- | --- |
| TexMaths12§display§\alpha§svg§600§FALSE§ | Shape parameter of ASV gamma distribution | alpha_dna | dnUniform |
| TexMaths12§display§sr§svg§600§FALSE§ | Vector of site rates | site_rates_dna |  |
| TexMaths12§display§\kappa§svg§600§FALSE§ | Transition/transversion rate | kappa | dnLognormal |
| TexMaths12§display§\pi§svg§600§FALSE§ | Base frequencies | pi | dnDirichlet |
| TexMaths12§display§Q§svg§600§FALSE§ | HKY instantaneous transition rate matrix | Q_DNA |  |
| TexMaths12§display§S§svg§600§FALSE§ | Phylogenetic continuous-time Markov chain | dna_seq | dnPhyloCTMC (clamped to cytochrome-b data) |

This module consists of a straightforward application of the HKY substitution model with four categories of among-site rate variation (ASV) from a discretised Gamma distribution.

The molecular data is an alignment of cytochrome-b gene sequences, extracted from the multilocus alignment of Pereira et al (2017). The use of a single locus was a compromise to improve the speed of the analyses.

# Brownian motion module

Figure 9. Visual representation of the script “morphology_brownian_multivariate.Rev”.

| Symbol | Meaning | Variable name | Distribution or value |
| --- | --- | --- | --- |
| TexMaths12§display§z§svg§600§FALSE§ | Multivariate Brownian process | zbrownian | dnPhyloMultivariateBrownianREML (clamped to log-ratio characters) |
| TexMaths12§display§VCV§svg§600§FALSE§ | Variance-covariance matrix | V |  |
| TexMaths12§display§R§svg§600§FALSE§ | Correlation matrix | R | Estimated with maximum likelihood |
| TexMaths12§display§\pi§svg§600§FALSE§ | Proportional rates | proportional_rates | dnDirichlet |
| TexMaths12§display§\zeta^2§svg§600§FALSE§ | Relative rates | relative_rates |  |
| TexMaths12§display§\alpha§svg§600§FALSE§ | Concentration parameter | alpha | 1.0 |

The evolutionary model for the log-ratio characters is Brownian motion, with the restricted likelihood parametrisation of Felsenstein (1973, 1981). We modelled the different rates of evolution and covariance between the log-characters with a variance-covariance matrix built with the “separation strategy” outlined in a RevBayes tutorial (May, 2019). The variance-covariance matrix is decomposed into an average change a vector of relative change rates and a correlation matrix. We assigned a Dirichlet prior with a concentration parameter of 1.0 to the relative rates vector. Because the Bayesian estimation of the 300 unique elements of the correlation matrix is be computationally prohibitive, we treated the correlation matrix as a fixed parameter, which we estimated from the data of extant species and the Pereira tree with restricted maximum likelihood fitting using Rphylopars v0.3.0 (Goolsby et al., 2016). 

# Results

In the traces of all the MCMC runs of model there were elements of the morphological branch rates vector and the relative Brownian rates vector that showed prolonged intervals where the Markov chain “flat-lined”, without accepting new moves (Fig. 10). Outside those intervals, the chains exhibited good mixing. In some instances the stalling of elements of the two parameters appeared in the same sample, but there is no apparent coincidence between parameters in the timing at which normal mixing is resumed.

The synchronicity of the stalling in different parameters suggests that complex parameter interactions might be at play. Yet, we have no satisfactory explanation for the stalling behaviour. We are unaware of the conditions that bring about these drastically different regimes of acceptance rates. It is possible that we our results come from a bug in RevBayes. That scenario merits further investigation.

Due to this aberrant behaviour of the MCMC, we interrupted the analyses and consider that the results cannot be given a meaningful biological interpretation.

Figure 10. Trace plots of elements of parameters of runs of model with uncorrelated exponential (UCE, plots A and B) and uncorrelated lognormal clocks (UCLN, plots C and D). Black arrows indicate where the chains stall and white arrows mark where they resume normal mixing. The samples shown here were taken every 1000 generations.

# References

Bouckaert R, Vaughan TG, Barido-Sottani J, Duchêne S, Fourment M, Gavryushkina A, Heled J, Jones G, Kühnert D, Maio ND, Matschiner M, Mendes FK, Müller NF, Ogilvie HA, Plessis L du, Popinga A, Rambaut A, Rasmussen D, Siveroni I, Suchard MA, Wu C-H, Xie D, Zhang C, Stadler T, Drummond AJ. 2019. BEAST 2.5: An advanced software platform for Bayesian evolutionary analysis. PLOS Computational Biology 15:e1006650. DOI: 10.1371/journal.pcbi.1006650.

Felsenstein J. 1973. Maximum-likelihood estimation of evolutionary trees from continuous characters. American Journal of Human Genetics 25:471–492.

Felsenstein J. 1981. Evolutionary trees from gene frequencies and quantitative characters: Finding maximum likelihood estimates. Evolution 35:1229–1242. DOI: 10.2307/2408134.

Gavryushkina A, Heath TA, Ksepka DT, Stadler T, Welch D, Drummond AJ. 2017. Bayesian total-evidence dating reveals the recent crown radiation of penguins. Systematic Biology 66:57–73. DOI: 10.1093/sysbio/syw060.

Goolsby EW, Bruggeman Jorn, Ané Cécile, Fitzjohn Richard. 2016. Rphylopars: fast multivariate phylogenetic comparative methods for missing data and within‐species variation. Methods in Ecology and Evolution 8:22–27. DOI: 10.1111/2041-210X.12612.

Höhna S, Heath TA. 2016. Phylogenetic inference using RevBayes: An introduction to calibrated time trees & divergence time estimation.

Höhna S, Heath TA, Boussau B, Landis MJ, Ronquist F, Huelsenbeck JP. 2014. Probabilistic graphical model representation in phylogenetics. Systematic biology 63:753–771.

Höhna S, Landis MJ, Heath TA, Boussau B, Lartillot N, Moore BR, Huelsenbeck JP, Ronquist F. 2016. RevBayes: Bayesian phylogenetic inference using graphical models and an interactive model-specification language. Systematic Biology 65:726–736. DOI: 10.1093/sysbio/syw021.

Joyce WG, Lyson TR. 2010. Pangshura tatrotia, a new species of pond turtle (Testudinoidea) from the Pliocene Siwaliks of Pakistan. Journal of Systematic Palaeontology 8:449–458. DOI: 10.1080/14772019.2010.500879.

Joyce WG, Parham JF, Lyson TR, Warnock RCM, Donoghue PCJ. 2013. A divergence dating analysis of turtles using fossil calibrations: an example of best practices. Journal of Paleontology 87:612–634. DOI: 10.1666/12-149.

King B, Qiao T, Lee MSY, Zhu M, Long JA. 2017. Bayesian Morphological Clock Methods Resurrect Placoderm Monophyly and Reveal Rapid Early Evolution in Jawed Vertebrates. Systematic Biology 66:499–516. DOI: 10.1093/sysbio/syw107.

Lewis PO. 2001. A likelihood approach to estimating phylogeny from discrete morphological character data. Systematic Biology 50:913–925. DOI: 10.1080/106351501753462876.

Lourenço JM, Claude J, Galtier N, Chiari Y. 2012. Dating cryptodiran nodes: origin and diversification of the turtle superfamily Testudinoidea. Molecular Phylogenetics and Evolution 62:496–507.

May MR. 2019.Multivariate Brownian Motion: Accounting for correlations among continuous traits. Available at https://revbayes.github.io/tutorials/cont_traits/multivariate_bm.html (accessed April 28, 2020).

Naksri W, Tong H, Lauprasert K, Suteethorn V, Claude J. 2013. A new species of Cuora (Testudines: Geoemydidae) from the Miocene of Thailand and its evolutionary significance. Geological Magazine 150:908–922. DOI: 10.1017/S0016756812001082.

Pereira AG, Sterli J, Moreira FRR, Schrago CG. 2017. Multilocus phylogeny and statistical biogeography clarify the evolutionary history of major lineages of turtles. Molecular Phylogenetics and Evolution 113:59–66. DOI: 10.1016/j.ympev.2017.05.008.

Thorne JL, Kishino H. 2002. Divergence time and evolutionary rate estimation with multilocus data. Systematic Biology 51:689–702. DOI: 10.1080/10635150290102456.
